# COVID-19 in People Living with HIV: A Systematic Review and Meta-Analysis

**DOI:** 10.3390/ijerph18073554

**Published:** 2021-03-30

**Authors:** Kai Wei Lee, Sook Fan Yap, Yun Fong Ngeow, Munn Sann Lye

**Affiliations:** 1 Department of Pre-Clinical Sciences, Faculty of Medicine and Health Sciences, Universiti Tunku Abdul Rahman, Kajang 43000, Malaysia; lee_kai_wei@yahoo.com (K.W.L.); ngeowyf@utar.edu.my (Y.F.N.); 2 Centre for Research on Communicable Diseases, Universiti Tunku Abdul Rahman, Kajang 43000, Malaysia; 3 Department of Population Medicine, Faculty of Medicine and Health Sciences, Universiti Tunku Abdul Rahman, Kajang 43000, Malaysia; lyems9@yahoo.com

**Keywords:** COVID-19, HIV, AIDS, systematic review, meta-analysis

## Abstract

COVID-19 is a global health emergency. People living with human immunodeficiency virus (PLHIV) have concerns about whether they have a higher risk of getting the infection and suffer worse COVID-19 outcomes. Findings from studies on these questions have largely been inconsistent. We aimed to determine the epidemiological characteristics, clinical signs and symptoms, blood parameters, and clinical outcomes among PLHIV who contracted COVID-19. Relevant studies were identified through Medline, Cinahl, and PubMed databases. A random-effects model was used in meta-analyses with a 95% confidence interval. Eighty-two studies were included in the systematic review and sixty-seven studies for the meta-analysis. The pooled incidence proportion of COVID-19 among PLHIV was 0.9% (95% CI 0.6%, 1.1%) based on the data from seven cohort studies. Overall, 28.4% were hospitalised, of whom, 2.5% was severe-critical cases and 3.5% needed intensive care. The overall mortality rate was 5.3%. Hypertension was the most commonly reported comorbidity (24.0%). Fever (71.1%) was the most common symptom. Chest imaging demonstrated a wide range of abnormal findings encompassing common changes such as ground glass opacities and consolidation as well as a spectrum of less common abnormalities. Laboratory testing of inflammation markers showed that C-reactive protein, ferritin, and interleukin-6 were frequently elevated, albeit to different extents. Clinical features as well as the results of chest imaging and laboratory testing were similar in highly active antiretroviral therapy (HAART)-treated and non-treated patients. PLHIV were not found to be at higher risk for adverse outcomes of COVID-19. Hence, in COVID-19 management, it appears that they can be treated the same way as HIV negative individuals. Nevertheless, as the pandemic situation is rapidly evolving, more evidence may be needed to arrive at definitive recommendations.

## 1. Introduction

The COVID-19 outbreak has been declared as a global health emergency by the World Health Organization [1]. Till date, the number of new COVID-19 cases has continued to increase daily in the backdrop of a lack of curative agents against the SARS-COV-2-virus. Vaccines have just been introduced but widespread implementation remains a significant issue. The clinical spectrum of COVID-19 ranges from mild through moderate to severe illness. Most infected individuals are asymptomatic or have a mild flu-like illness. Moderate and severe illness present with features of lower respiratory tract involvement that can proceed to respiratory failure, shock, and multiple organ failure, which can be fatal [2]. Common laboratory abnormalities of the infection are markers of systemic inflammation and coagulopathies, reflections of the underlying pathologies and complications of serious illness [2,3,4].

It is widely known and reported that older people, people who are immunocompromised, and those with underlying co-morbidities such as hypertension, diabetes mellitus, and pre-existing respiratory and cardiovascular disorders are at significantly higher risk of severe illness that requires hospitalization and intensive supportive therapy and care [5,6]. For example, people with hypertension had a significantly higher mortality risk from COVID-19 (pooled odds ratio = 3.36) compared with the normotensive population [7]. A meta-analysis reported that the odds ratio for mortality among those with diabetes was 1.75 (*p* < 0.01) [8]. Patients with chronic obstructive pulmonary disease had higher risk for severe COVID-19 (pooled relative risk = 1.88, *p* < 0.05) [9]; likewise those with cardiovascular disease (odds ratio = 4.85, *p* < 0.05) [10] compared to those without these comorbidities. 

A systematic review reported that among people living with human immunodeficiency (PLHIV) who contracted COVID-19, the most common co-morbidities were hypertension (39.3%) and obesity (19.3%) [11]. More than half (66.5%) had mild-moderate symptoms, 74% had fever, and 58.3% had cough [11]. Another review indicated that PLHIV with well-controlled disease were not at higher risk for poorer COVID-19 outcomes than the general population [12]. However, these findings cannot be extrapolated to all PLHIV as these reviews are limited by small sample size and the disproportionate number of symptomatic COVID-19 cases who required hospitalization compared to those with mild or no symptoms of COVID-19. Further, the pooled incidence of COVID-19 and disease manifestations among PLHIV remains unknown. PLHIV often have concerns and questions related to their risk of serious illness from COVID-19 [13]. The limited availability of data emphasises the need for more studies to address these concerns. To date there is no specific treatment for COVID-19; patient management is largely supportive and symptomatic including the use of drugs to mitigate the complications of the infection [14,15].

In this rapidly evolving situation, clinicians and scientists are still learning about COVID-19 and how it affects PLHIV. Although the clinical characteristics of COVID-19 have been broadly described [16], there is still scarce literature on the clinical features including comorbidities, presenting symptoms and signs, severity, complications, and outcomes among PLHIV who contracted the infection. There are also uncertainties regarding the laboratory parameters that are predictive of disease severity and outcome of COVID-19 among PLHIV. Likewise, it is unclear whether factors related to the HIV infection and its management as well as patient compliance influence the clinical presentation, severity, and outcome of COVID-19. To date, the information reported in the literature has been inconsistent. We therefore conducted a systematic review and meta-analysis to determine the incidence proportion of COVID-19 among PLHIV and review the clinical course and outcomes in PLHIV co-infected with COVID-19. In addition, we also compared the clinical and laboratory features, and disease outcome between PLHIV who are on highly active antiretroviral therapy (HAART) and those who are not. 

## 2. Materials and Methods

We conducted this review according to Preferred Reporting Items for Systematic Reviews and Meta-Analyses (PRISMA) (Supplementary Table S1) [17]. This study has been registered with PROSPERO (registration number: CRD42020210161).

### 2.1. Literature Search

Three databases (Medline, Cinahl, and PubMed) were searched on 20 September 2020 by two investigators independently (KWL and SFY) to identify potential studies. The search strategy consisted of several terms which were: (HIV OR human immunodeficiency virus* OR AIDS OR acquired immunodeficiency syndrome) AND (COVID-19 OR coronavirus OR 2019-NCOV OR nCoV* OR COVID* OR SARS-CoV*) with limiters of ENGLISH and HUMAN (Supplementary Table S2). We performed reverse and forward citation tracking; the last search was done on 26 October 2020. Papers published beyond this date were not considered for inclusion into this review. 

### 2.2. Data Handling

All relevant articles identified through the above databases were imported into the Endnote^®^ programme version X5, after which de-duplication was performed. Subsequently, titles and abstracts were reviewed for their relevance by two clinical pathologists (SFY and YFN) and the full texts of the selected articles were assessed for their eligibility to be recruited into this systematic review and meta-analysis. 

### 2.3. Selection Criteria

Prospective and retrospective cohort studies, cross-sectional studies, case-control studies, case series, and case reports were eligible for inclusion. Studies were excluded if they were randomized controlled trials, controlled clinical trials or any type of review and meta-analysis. The studies must have data on the number of COVID-19 cases among HIV/AIDS patients or possess any data related to comorbidities, HIV profile, pharmacological treatment and supportive care for COVID-19, and laboratory test results. In addition, presenting symptoms and signs, imaging findings, and contact history were used as supplemental information for the evaluation of the diagnoses. We also examined the clinical outcomes of COVID-19, and the cause of death among those with HIV/AIDS infected with COVID-19. Any disagreements among the investigators were resolved through discussions and consultations with the third senior investigator (MSL) before the final consensus for quantitative analysis was reached. 

### 2.4. PICO

The participants should be HIV patients (age > 18 years) with a confirmed diagnosis of COVID-19 by nucleic-acid based testing, or with positive serological test and/or presence of typical radiological findings and clinical features of COVID-19. Exposure was referred to as COVID-19 infection. There was no comparator in the current review. The main outcomes for this review were pooled percentage (or pooled incidence proportion) of COVID-19 among those with HIV infection and the mortality rate due to COVID-19 among HIV-infected people. Secondary outcome was pooled prevalence of symptoms among HIV patients diagnosed with COVID-19. 

### 2.5. Data Extraction 

This step was performed independently by two investigators (KWL and SFY) and proofread by another two investigators (YFN and MSL). The information we extracted from the included studies were basic characteristics of studies as well as demographic characteristics, severity of disease, and mortality rate among patients with HIV-COVID-19 co-infection; data on comorbidities and lifestyle-related disorders, HIV profiles, diagnostic methods for COVID-19, vital signs, findings of chest imaging, pharmacological and supportive care given to treat the disease, and symptoms experienced by HIV-COVID-19 patients throughout the clinical course as well as the results of laboratory investigations (inflammation markers, liver profile, serum creatinine, serum lactate dehydrogenase, D-dimer, and full blood counts). 

### 2.6. Quality Assessment 

The quality of the papers included was independently assessed by two investigators (KWL and SFY). We used the checklist “Strengthening the Reporting of Observational Studies in Epidemiology” (STROBE) to assess the quality of cohort studies [18]. We used the quality appraisal checklist developed by the Institute of Health Economics to appraise individual studies pertaining to case series or case reports (Supplementary Table S3) [19]. 

### 2.7. Operational Definition

Advanced HIV/AIDS infection for adults is defined as those with clinical stage 3 or stage 4 disease or where CD4 count is available, with CD4 < 350 cells/mm^3^ at any clinical stage [20]. Undetectable viral load is defined as <20 RNA copies/mL. We used severity of COVID-19 as defined by the World Health Organization [21] (Supplementary Table S4). In brief, mild disease is defined as symptomatic disease meeting the case definition for COVID-19 with no evidence of viral pneumonia or hypoxia. Moderate disease is defined as symptomatic disease with clinical signs of pneumonia (such as fever, cough, dyspnoea, and increased rate of breathing) but without features of severe pneumonia. Severe disease is defined by clinical signs of pneumonia that are accompanied by a respiratory rate > 30 breaths/min, or oxygen saturation less than 90% (SpO_2_ < 90) while breathing room air, or clinical symptoms of severe respiratory distress. Critical disease is defined as a disease that is complicated by either acute respiratory distress syndrome or sepsis/septic shock. 

### 2.8. Data Syntheses

For meta-analysis, the incidence proportion of COVID-19 among PLHIV was derived from cohort studies that had inception cohorts of HIV patients without COVID-19 at baseline. For symptoms presented by patients with HIV-COVID-19 co-infection, data were derived from all cohort studies and case series with sample size not less than 30 people for meta-analysis. The results were pooled only if comparable data were available from more than two studies. Meta-analysis was performed with Open Meta (Analyst)^®^ [22] using a random-effect model (DerSimonian and Laird method) to produce the pooled incidence proportion (PIP) or prevalence and their respective 95% CIs. Heterogeneity was assessed using I^2^, with a *p*-value of less than 0.05 as significant. A sensitivity analysis was conducted using leave-one-out meta-analysis to examine how the exclusion of each individual study affects the overall estimate of the rest of the studies. 

## 3. Results

### 3.1. Search Results

**Figure 1:**
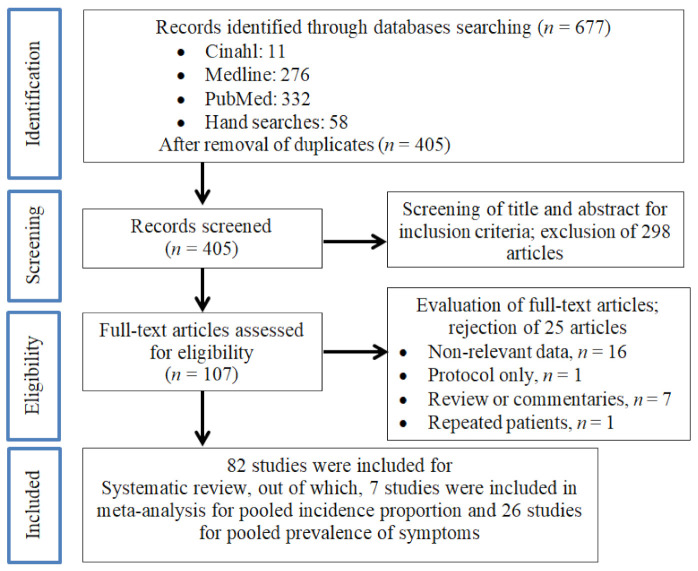
Preferred Reporting Items for Systematic Reviews and Meta-Analyses (PRISMA) flow diagram of the literature screening process.

We identified 677 articles in the initial screening, as shown in Figure 1. After the removal of duplicate articles (*n* = 272), a total of 405 articles were retrieved for further assessment. After screening for suitability through the title and abstract, 107 articles were selected for full-text assessment. After careful evaluation, 82 articles were finally included for the systematic review, out of which, 7 cohort studies were deemed eligible for meta-analysis for PIP of COVID-19 among PLHIV and 26 studies (24 cohort studies and 2 case series) for pooled prevalence of symptoms. Only three studies [23,24,25] were used simultaneously for the systematic review and meta-analysis for PIP and pooled prevalence of symptoms. 

### 3.2. Description of Included Studies 

The main characteristics of the 82 studies are summarized in Table 1. A total sample of 643,018 HIV patients from 19 countries was included in the analysis. Twenty-four of these studies were in the USA, 14 in China, 12 in Italy, 5 in Spain and the UK, 4 in South Africa, and 3 in Japan. Of the remaining, two each were in Brazil, France, and Germany, and one each from Cyprus, India, Libya, Panama, Peru, Singapore, South Korea, Turkey, and Uganda. Out of 82 studies, 45 were case series, 26 studies were case reports and 11 were cohort studies. 

### 3.3. Incidence Proportion of COVID-19 among HIV Patients and Their Demographic Characteristics, Severity and Mortality

**Figure 2:**
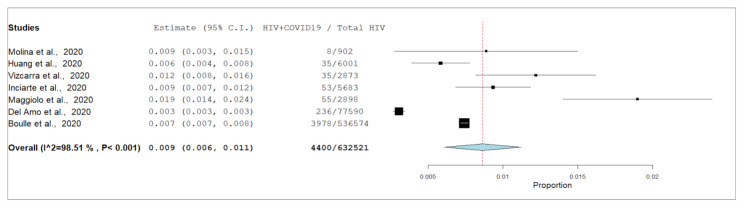
Pooled incidence proportion of COVID-19 among PLHIV based on data accumulated from cohort studies.

The PIP of COVID-19 among PLHIV was 0.9% (95% CI 0.6%, 1.1%, I^2^ = 98.5%) based on the data gathered from seven cohort studies (Figure 2). The mean age of PLHIV co-infected with COVID-19 was 49.3 years ± 10.7. More than half (*n* = 3645) were female (58.5%), 2483 (39.8%) were male, 94 were of unknown gender (1.5%), and 13 (0.2%) were transgender. The mean body mass index (BMI) was 25.8 ± 3.8 (*n* = 261; range = 18.1–31.5). The severity of COVID-19 was known in 347 cases, of which 7.8% (27/347) were critical, 36.3% were severe (126/347), 32.8% (114/347) were moderate, and 23.1% (80/347) were mild. Out of a total of 6235 cases, 1773 (28.4%) needed hospitalization, of whom 2.3% (218/1773) were severely ill and were admitted to the intensive care unit. The overall mortality rate among PLHIV co-infected with COVID-19 was 5.3% (328/6235).

### 3.4. Comorbidities among PLHIV Co-Infected with COVID-19

As shown in Supplementary Table S5, cardiovascular disease (27.2%) was the most frequent comorbidity, of which hypertension accounted for 23.9%. This was followed by diabetes mellitus (12.2%), chronic lung disease and asthma (4.2% each), and lifestyle-related disorders comprising dyslipidaemia and obesity (5.4%), smoking, alcoholism, and illicit drug intake (2.9%, 0.7%, and 1.3% respectively). Other comorbidities of note are chronic kidney (6.4%) and liver disorders (1.4%).

### 3.5. HIV Profile of PLHIV Co-Infected with COVID-19

The diagnosis of HIV was made between 1988 and 2020; the mean duration of infection ranged from under 1 year to 30 years. Anti-retroviral treatment (ART) was documented in 1253 out of 6235 (20.1%) of cases, with a mean duration of receiving antiretroviral treatment ranging from 2 to 23 years. Ten patients were newly diagnosed, thus treatment naïve, and another 9 patients were not on treatment for unspecified reasons. Nucleoside reverse transcriptase inhibitor (NRTI) was the most frequently prescribed ART for the HIV patients, followed by integrase strand transfer inhibitor, non-nucleoside reverse transcriptase inhibitors, and protease inhibitors.

The CD4 cell counts before diagnosis of COVID-19 were available in only 844 out of 6235 cases; the nadir CD4 cell count ranged from 97 to 434 in these cases. Out of the 844 cases, 115 had counts of <350 cells/uL while the remaining 729 patients had counts of ≥350 cells/uL.

Using the World Health Organization (WHO) HIV/AIDS revised clinical staging [20], 354 out of 6235 (5.7%) patients met the criteria for advanced disease, 115 of which were defined by immunological criteria (CD4 cell count <350 µL), and 239 by clinical criteria (clinical Stage 3 or 4). Results on the viral load measurements pre-COVID was available in 1023 HIV patients of whom 294 had undetectable viral load (<20 RNA copies/mL) and 729 had >20 RNA copies/mL. Details on the HIV profiles are provided in Supplementary Table S6.

### 3.6. Vital Signs, Results of COVID-19 Testing and Chest Imaging Findings

The majority of studies (*n* = 5662 patients) used nucleic acid-based testing to diagnose COVID-19. A total of 5522 patients (97.5%) tested positive. In 125 patients (2.2%), the results of nucleic acid testing were not known, and 15 cases (0.3%) tested negative. Interestingly, six cases negative for the nucleic acid-based test were seropositive for antibodies to COVID-19. The method to diagnose COVID-19 was not known in 496 cases, of which 87 were reported as positive cases. Another observation is that 46 cases had at least one negative result (up to maximum 7 negative results) prior to conversion to positivity for the nucleic acid-based test despite the presence of multiple symptoms related to the infection.

COVID-19 antibody (IgM and/or IgG) testing was done in 89 patients. The results demonstrated that some cases positive for the virus nucleic acid either remained persistently seronegative (*n* = 11) or had a prolonged viral shedding duration. In the latter, 8 cases who were IgM positive continued viral RNA shedding from beyond day 14 up to 41 days, while 6 cases who were IgG positive continued to shed from 15 to 32 days.

On presentation, the body temperature ranged from 35.9 to 40 °C and the systolic and diastolic blood pressure from 93 to 172 mmHg and 33 to 97 mmHg, respectively. Pulse rate and respiration rate ranged from 57 to 160 beats/minute and 14 to 40 breaths/minute, respectively. Oxygen saturation when breathing ambient air ranged from 56 to 100 mmHg; in 27 cases, the oxygen saturation was below 90% (hypoxaemia). PaO_2_/FiO_2_ ratio ranged from 50 to 500 mmHg; about 50% of the cases had mild to severe hypoxemia (refer to Supplementary Table S7 for details).

### 3.7. Treatment for COVID-19

Details of the treatments given to PLHIV co-infected with COVID-19 are given in Supplementary Table S8. Information regarding pharmacological intervention was recorded in 1429 patients. Hydroxychloroquine was given to 413 (28.9%) patients. Eighty-three patients received anti-virals which included Oseltamivir, Arbidol, or interferon-alpha (*n* = 41, 2.9%) and Remdesivir (*n* = 27, 1.9%). Various antibiotics were prescribed for 346 (24.2%) patients for prevention or treatment of secondary bacterial infections.

Symptomatic treatment including anti-inflammatory agents (steroids, IL-6 inhibitor—sarilumab) and/or convalescent plasma was recorded for 167 (11.7%) cases. Anticoagulant therapy (Enoxaparin sodium or Heparin) was given to 15 (1%) patients. Fifty (3.5%) patients did not receive any pharmacological treatment.

Information on the use of supportive care and therapy was available in 1054 patients. Of these, 352 patients needed supplemental oxygen via venturi or face masks; 211 patients required non-invasive mechanical ventilation (bi-level positive airway pressure—BiPAP, or continuous positive airway pressure—CPAP) and 144 patients needed invasive ventilation, of whom, three were given extracorporeal membrane oxygenation (ECMO).

Information on the status of treatment for HIV while combating COVID-19 was available in 206 cases. The treatment for HIV was maintained for 190 patients, while in 8 patients, it was modified and in the last 8 patients, it was discontinued temporarily.

### 3.8. Symptoms of COVID-19

Results from studies on COVID-19 symptoms experienced by PLHIV throughout their illness are detailed in Supplementary Table S9. The symptoms that were analyzed in the current review and the results for the pooled prevalence of symptoms are as summarized in Table 2. Meta-analysis showed that fever was the most common symptom (79.1%, 95% CI 65.8–76.4) followed by dry cough (66.3%, 95% CI 58.0–74.7) and dyspnoea (46.0%, 95% CI 34.3–57.6). General symptoms such as lethargy and myalgia were also quite frequent (33.6%, 95% CI 20.6–46.7; and 28.1%, 95% CI 18.7–37.5 respectively). Less frequent but widely recognised symptoms were gastrointestinal upsets, anosmia and ageusia, chest discomfort, and mental changes. Overall, the heterogeneity between the pooled studies was relatively high, with I^2^ greater that 75% in half the cases (Forest plots are for the symptoms are shown in Supplementary Figure S1–S16).

### 3.9. Blood Parameters among PLHIV Co-Infected with COVID-19

Data on the blood parameters which include inflammatory markers, serum enzymes, serum creatinine and haematological parameters are presented in Table 3. Generally, a fairly high percentage of studies reported elevated baseline values in most of the inflammatory markers (C-reactive protein, fibrinogen, ferritin, and interleukin-6), markers of clotting disorder (D-dimer) and markers of cellular/tissue damage (in particular lactate dehydrogenase). The percentage of cases with elevated biomarkers indicative of inflammation, coagulopathy, and tissue damage was correspondingly high. The results of the liver profile were fairly variable across studies and the effect of COVID-19 on these analytes was inconsistent. The mean serum creatinine level was within the reference limits in over two thirds of the studies. Haemoglobin and leukocytes were normal in most of the patients while lymphocytes were low in about 50% of cases.

### 3.10. Subgroup Analysis of PLHIV on HAART Compared with Those Who Were Not

Results of subgroup analysis on selected laboratory findings and clinical features of PLHIV co-infected with COVID-19 who (i) were on HAART and compliant with the treatment (*n* = 19) and (ii) those who were not—reason unspecified (*n* = 9) are shown in Table 4.

Those who were on HAART had a mean CD4 count of 556.9 (range = 201–1827) while those who were not had a mean count of 331.8 (range = 10–504). As expected, the mean viral load among those on HAART was significantly lower with a mean of 24.7 (range = 20–40 RNA copies/mL) compared to who were not (mean = 50,493; range = 20–293,313/mL). These results support a more serious HIV infection in those not on HAART, regardless of the reason, and by inference a greater degree of cellular immune-deficiency, which is supported by the HIV disease classification based on clinical criteria [105].

However, the clinical presentation of COVID was quite similar in both groups of HIV patients; likewise, disease severity as judged by the number of ICU admissions. Similarly, the acute inflammatory markers, D-dimer and lactic dehydrogenase, were elevated in both groups of HIV individuals to a similar extent. The apparent higher levels of D-dimer and ferritin among those on HAART and higher levels of IL-6 and lactic dehydrogenase among those not on HAART are of no consequence in view of the very small number of cases with these results.

## 4. Discussion

The purpose of this study is to systematically review and conduct meta-analysis using data from current studies reporting on epidemiological characteristics and outcomes, and the clinical course in PLHIV co-infected with COVID-19. A limited comparison of clinical features, laboratory findings, and disease severity (based on hospitalization, admission to ICU and death) between those on HAART and those who were not was also included.

### 4.1. Epidemiology, Comorbidities, and Outcomes of COVID-19 in PLHIV

In the current review, the overall PIP of COVID-19 among PLHIV from meta-analysis was 0.9% (95% CI 0.6%, 1.1%; I^2^ = 98.5%). To address the high degree of heterogeneity across the seven cohort studies used for the estimation of the overall PIP of COVID-19, leave-one-out meta-analysis was performed. The results demonstrated that each of these studies had substantial effect (*p*-value < 0.001) on the overall estimate of the rest of the studies (PIP varying from 0.7 to 1.0). (Supplementary Table S10: Summary of estimates and its 95% CI from leave-one-out Meta-analysis; Supplementary Figure S17: Leave-one-out Meta-analysis forest plot). The heterogeneity between these studies is further demonstrated by the forest plot (Figure 2) and by the I^2^ result and the corresponding *p*-value. The incidence proportion between studies ranged from 0.3% (Del Amo et al.) [30] to 1.9% (Vizcarra et al.) [88]. Methodological variations such as sampling methods, study design and study size (range: 902 to >530,000 PLHIV) are noted as possible contributary factors for this finding.

Overall, 28.4% of PLHIV affected by COVID-19 were admitted to hospital. The infection was classified as severe-critical in 2.5% of patients and 3.5% of patients were admitted to the intensive care unit. The mortality rate was 5.3%. Severe or critical COVID-19 patients were older (54.8 years ± 13.2) compared to non-severe/critical cases (46.7 years ± 11.8). Further, among the severe/critical cases, the frequency of comorbidities was much higher compared to the non-severe/critical cases. For example, hypertension and diabetes were present in 25% and 10% in the former compared to 13.9% and 7.6%, respectively, in the latter group. These results are consistent with those reported in previous reviews in which older age and the presence of comorbid conditions, in particular hypertension or diabetes, were associated with severe COVID-19 [106].

The most common comorbidity among all PLHIV suffering from COVID-19 was hypertension (23.9%) followed by diabetes (12.2%). Previous reviews have similarly reported a high prevalence of hypertension (14.3% to 27.4%) and diabetes (7.7% to 17.4%) in the general population infected with COVID-19 [5,107,108,109,110]. In another review on the prevalence of comorbidities among fatal COVID-19 cases, hypertension was also the most common (38.6%) followed in decreasing frequency by diabetes (22.2%), chronic cardiovascular disease (17.5%), and cerebrovascular disease (15.6%) [111]. Pranata et al. reported that hypertension was associated with severe COVID-19 (risk ratio 2.04), intensive care unit admissions (risk ratio 2.11), and mortality (risk ratio 2.11) [112]. These observations clearly demonstrate that hypertension is the most prevalent comorbidity among people with COVID-19 and is also a risk factor for adverse outcomes.

This is not surprising as hypertension is one of the most common chronic diseases with a global prevalence of approximately 31.1% in the year 2010 [113]. Another possible explanation for hypertension being a risk factor for severe outcome and mortality could be that hypertensive patients have increased angiotensin-converting enzyme 2 expression [114] and this increases the risk of the patient to severe COVID-19 as more of the SARS-CoV-2 virus can bind with the increased number of ACE2 receptors in the lungs to enter cells [115]. A point of note in this regard is that hypertensive patients are often treated with angiotensin-converting enzyme inhibitors and angiotensin receptor blockers. However, some studies have shown that these pharmacological treatments increase angiotensin-converting enzyme 2 expressions [116,117], thereby facilitating the entry of SARS-CoV-2 to cause severe outcomes and mortality [118].

It is noteworthy that lifestyle related disorders appear to be quite common, in particular obesity and dyslipidaemia, followed by smoking and illicit drug intake, the last not unexpected, being a risk factor for HIV infection.

### 4.2. Chest Imaging Findings in PLHIV Co-Infected with COVID-19

Chest imaging examinations are useful in monitoring hospitalised patients particularly those with moderate to severe symptoms and those at risk of progression, as well as for the evaluation of complications. Further, they can be useful for medical triage of patients with moderate or severe symptoms in settings with limited resources for nucleic acid-based diagnosis, and act as prompts for clinicians to be cautious with patients who might have COVID-19 despite repeated negative nucleic acid-based test results. It is noted, however, that chest imaging findings are quite varied and dependent on the stage and severity of the illness, and underlying comorbidities [119,120].

In this review, the more common abnormalities of clinical importance reported were ground-glass opacities (GGO), consolidation, and reticulation. Of these, GGO, widely recognised as an early and typical feature, was the most frequent abnormality observed, being found in 72.2% of patients with mild-to-moderate disease, and 68.8% of patients with severe-critical disease. The distribution of ground glass infiltrations was predominantly bilateral, which is similar to that described in several reviews [121,122,123,124].

Apart from GGO, consolidation, and reticulation, the wide variety of other imaging findings that were reported by the various studies in this series was similar to that reported, albeit at varying frequencies, in COVID-19 patients in general. Finally, in most patients with either bilateral (75%) or unilateral pneumonia (25%), chest imaging manifestations were similar regardless of COVID-19 severity.

### 4.3. Clinical Features in PLHIV Co-Infected with COVID-19

The most common symptom in PLHIV co-infected with COVID-19 was fever (71.1%), a finding broadly consistent with previous reviews which reported an overall frequency ranging from 85.6% to 91.3% [5,107,122]. In a separate review on COVID-19 in cancer patients [115], fever was found to be present in 85.4% of cases. Less frequently reported symptoms include headache (17.9%), altered mental status (8.0%), anosmia (11.5%), and ageusia (9.7%). The prevalence of these relatively uncommon symptoms is highly variable across previous reviews [125,126,127] giving rise to concerns. For example, the reported frequency of impaired smell sensation varied between 8.3% and 59.9% and that for impaired taste sensation varied between 6.7% and 57.5%. The reported frequency of impaired consciousness was between 1.6 and 5.0%. While headache may be multifactorial in its aetiopathogenesis, anosmia, ageusia, and altered mental status are clearly neurological in origin, and thus of clinical interest and relevance.

SARS-COVID-2 has been known to exhibit neurotrophic properties and can directly infect the nervous system [128]. The ACE2 receptors which are the cellular entrance for this virus have been found to be abundant not only on epithelial cells of the tongue [129] and lungs [130] but also on glial cells and neurons. Therefore, the virus could possibly gain entry into the central nervous system via a neuronal pathway or neuronal transport, thereby causing damage to nerve functions [131]. Chilvers et al. reported that the virus is able to disrupt nasal epithelium and is released on the apical and basolateral side of epithelial cells to reach the central nervous system via the bloodstream [132]. However, the exact process by which olfactory and gustatory dysfunctions and damage occur remains to be elucidated. Based on the neurotrophic potential of SARS-CoV-2, some have suggested the possibility that these neurological symptoms might persist even after patients have recovered from COVID-19 [133], but to-date, there is still no evidence to support this assumption.

### 4.4. Blood Parameters in PLHIV Co-Infected with COVID-19

Most studies [111,134] have demonstrated an abnormal elevation of various inflammation markers in COVID-19 patients, which could be indicative of a hyper-inflammatory state and poor disease prognosis.

The changes in white blood cell counts in COVID-19 are variable depending on the clinical phase of the illness. Generally, lymphopenia, which is reported in many studies, is evident with the onset of symptomatic disease during which the systematic inflammatory response or even the “Cytokine storm” may occur in more severe cases. This observation is partially ascribed to the presence of ACE2 receptors on the surface of lymphocytes allowing direct injury by the SARS-CoV-2 virus. Alternatively, the release of inflammatory markers including interleukins, granulocyte colony stimulating factor (GCSF) and tumor necrosis factor alpha (TNF-α) may induce apoptosis of the cells. Hence, the observed association between lymphopenia and severity or poor prognosis of the illness. Indeed, in the present review, lymphopenia was present in an average of 42.6% of symptomatic individuals, compared to 20% of asymptomatic individuals. Other findings of note are increased neutrophils in 37.5% and decreased platelets in 39.1% of symptomatic cases. However, sequential changes in blood counts were not documented in most studies on PLHIV with COVID-19. In this review, we found only three studies which reported changes in full blood counts [55,81,102]; however, all three reported cell counts within the normal range on admission and throughout hospitalization. While this may be the case, it is not unreasonable to expect that PLHIV will demonstrate a blood cell profile largely similar to that of non-HIV infected people as a response to the infection.

### 4.5. Comparison between PLHIV with COVID-19 on HAART and Those Who Were Not

Immunocompromised individuals are expected to be more highly susceptible to severe COVID-19 [135]. However, this general assumption was not borne out by the results of our study. In our subgroup comparison, PLHIV who were on the HAART regimen had higher CD4 cell counts, and lower HIV viral loads compared to those who were not, as expected. Logically, the former group should have better cellular immunity than the latter. However, subgroup analysis did not demonstrate any relevant difference with respect to the clinical features, laboratory findings, and severity of illness. It is acknowledged, however, that the number of subjects with data for this sub-group analysis was too small to provide any definite conclusions.

### 4.6. What Are the Impacts of COVID-19 on Health Care for People with HIV?

The rapid spread and exponential increase in the number of COVID-19 infections globally has strained healthcare systems due to the diversion of resources from less urgent services to COVID-19 control and management [136]. This would be expected to impact negatively the delivery of care for other illnesses. With specific reference to HIV infection, this could result in disruption of the broad spectrum of care including the initiation and maintenance of ART [137,138,139]. In the broader perspective, the UNAIDS’ first 90-90-90 target for 2020 will be challenged [140]. Research and development funding and activity have also been diverted to or focussed on COVID-19 [141]. However, it should be noted that measures have been taken to minimise these negative effects through streamlining of processes and the use of the internet [142].

### 4.7. Is COVID-19 Vaccine Safe for PLHIV?

Currently, several types of vaccines including mRNA, viral vector, inactivated whole virus, and protein subunit vaccines are in phase 3 clinical trials, some of which have been approved for emergency use to protect against serious complications of the infection. Of these different vaccines, data on the trials that included PLHIV are relatively limited. These include trials that involved the Pfizer/BioNTech (BNT162b2) mRNA vaccine [143], the Moderna mRNA (mRNA-1273) vaccine [144], the Oxford-AstraZeneca viral vector vaccine (AZD1222) [145], and the Janssen viral vector vaccine (Ad26.COV2.S) [146]. The reported efficacy in terms of preventing symptomatic illness, and moderate as well as severe/critical illnesses ranged from 66% to 95% [145,146,147,148]. The data on safety of the vaccines are also limited due to the short time frame since vaccination studies commenced. Of note is that the numbers of PLHIV included in these studies were relatively small and data on their response to vaccination were not separately reported. This underlies the need for more studies to address this question. It is also noted that the emergence of new strains of the SARS-CoV-2 virus will further complicate the picture.

### 4.8. Does COVID-19 Affect Patients with Long-Term HIV More?

Data on the severity of COVID illness among PLHIV in relation to the duration of HIV infection was available in a small number of cases. Based on arbitrary definition for long-term HIV (diagnosed with HIV for at least 8 years or longer), 71 cases were considered as long-term HIV patients and 40 as short term. Briefly, 41 (57.7%) long-term HIV infected cases had severe/critical COVID illness compared to 20 (50.0%) of those with short-term HIV infection while the admission rate to ICU was 26.7% and 7.5%, respectively. From the foregoing data, it would appear that long-term HIV infection is associated with more severe illness; however, the significance of these figures needs to be established with further studies.

### 4.9. Strengths and Limitations

This review contributes to existing knowledge of COVID-19 infection in PLHIV by providing a comprehensive investigation of epidemiological characteristics, clinical signs and symptoms, blood parameters, and clinical outcomes based on a large sample size of more than 6000 PLHIV co-infected with COVID-19. Being limited by the unavailability of some data and the small sample size in some studies, we were unable to conduct a meta-analysis for all parameters. In the subgroup comparison (patients on HAART versus patients not on HAART), there were few patients in both subgroups (19 and 9 cases, respectively). As for the prevalence of asymptomatic patients, it was unclear how these patients were detected. This could be dependent on the community and environment in which the PLHIV lived and the intensity of the detection effort by the health authorities. As such, there could be a considerable degree of detection bias giving rise to unreliable estimates of the proportion of asymptomatic cases in the study.

## 5. Conclusions

This study has shown a COVID-19 pooled incidence proportion of 0.9% (95% CI 0.6%, 1.1%) among PLHIV and 5.3% of mortality for PLHIV co-infected with COVID-19. Hypertension and fever were the most common comorbidity and symptom, respectively. Most inflammation markers were elevated on admission. As the C0VID-19 pandemic is still rapidly evolving, further studies are needed to corroborate the results of this review. Considerably more data will need to be analyzed particularly for the comparison of PLHIV with COVID-19 on HAART and those who are not.

## Figures and Tables

**Table 1:** Characteristics of the included studies.

No	Author	Year	Country	Study Design	Quality Assessment	HIV + COVID-19	Total HIV	HIV + COVID-19 Patients Characteristics				Summary of Severity of Infection at Admission (%)	Number of HIV + COVID-19 Hospitalized (%)	Number of Hiv + COVID-19 Admitted to Intensive Care Unit (%)	Number of Deaths among HIV + COVID-19 (%)
								Average Age	Average BMI	Gender, *n* (%)	Ethnicity				
1	Adachi et al. [26]	2020	Japan	CS	Supplementary Table S3	2	2	38.7		Transgender women, 2 (100)			2 (100)		
2	Altuntas et al. [27]	2020	Turkey	CS	Supplementary Table S3	4	1224	43.5		Male, 4 (100)			4 (100)	1 (25)	1 (25)
3	Baluku et al. [28]	2020	Uganda	CR	Supplementary Table S3	1	1	34.0		Female, 1 (100)			1 (100)		
4	Benkovic et al. [29]	2020	USA	CS	Supplementary Table S3	4	4	50.5		Male, 4 (100)			1 (25)		
5	Blanco et al. [30]	2020	Spain	CS	Supplementary Table S3	5	5	45.9		Male, 3 (60); Transgender, 2 (40)		Severe, 2 (40); Moderate, 1 (20); Mild, 2 (40)	5 (100)	2 (40)	
6	Boulle et al. [31]	2020	South Africa	RC	Good	3978	536,574			Male, 877 (22); Female, 3101 (78)			601 (15.1)		115 (2.9)
7	Byrd et al. [32]	2020	USA	CS	Supplementary Table S3	27	27	53.0		Male, 21 (77.8); Female, 5 (18.5); Transgender, 1 (3.7)	Hispanic, 13; African American, 6; White, 7; Other, 1		9 (33.3)		1 (3.7)
8	Calza et al., b [33]	2020	Italy	CS	Supplementary Table S3	14	14	52.6		Male, 9 (64.3); Female, 5 (35.7)	White, 13; Other, 1		3 (21.4)		
9	Calza et al., a [34]	2020	Italy	CS	Supplementary Table S3	26	26	53.8		Male, 19 (73.1); Female, 7 (26.9)	Caucasian, 25; Unknown, 1		5 (19.2)		
10	Chen et al. [35]	2020	China	CR	Supplementary Table S3	1	1	24.0		Male, 1 (100)			1 (100)		
11	Chiappe et al. [36]	2020	Peru	CR	Supplementary Table S3	1	1	38.0		Male, 1 (100)					1 (100)
12	Childs et al. [37]	2020	UK	CS	Supplementary Table S3	18	18	52.0		Male, 12 (66.7; Female, 6 (33.3)	Black, 17; Non-black, 1		18 (100)		5 (27.8)
13	Cipolat and Sprinz [38]	2020	Brazil	CR	Supplementary Table S3	1	1	63.0		Female, 1 (100)		Severe, 1 (100)	1 (100)		
14	Coleman et al. [39]	2020	UK	CR	Supplementary Table S3	1	1	55.0		Male, 1 (100)			1 (100)	1 (100)	
15	Collins et al. [40]	2020	USA	CS	Supplementary Table S3	20	20	57.0	28.0	Male, 13 (65); Female, 6 (30); Transgender, 1 (5)	American African, 17; White, 1; Mixed race 1; Hispanic/Latino1		20 (100)		
16	Dandachi et al. [41]	2020	USA	CS	Supplementary Table S3	286	286	51.4		Male, 212 (74.1); female; 74 (25.9)	African American, 133, Hispanic, 78; White, 48; Asian, 21; Unknown, 6		164 (57.3)	47 (16.4)	27 (9.4)
17	Del Amo et al. [42]	2020	Spain	Cohort	Good	236	77,590			Male, 204 (86.4); Female, 32 (13.6)			151 (64)	15 (6.4)	20 (8.5)
18	Di Biagio et al., a [43]	2020	Italy	CS	Supplementary Table S3	4	1500	66.6		Male, 3 (75); Female, 1 (25)			4 (100)		
19	Di Biagio et al., b [44]	2020	Italy	CS	Supplementary Table S3	69	69	53.5		Male, 50 (72.5); Female; 19 (27.5)	Caucasian, 59; Others, 10	Moderate-Severe, 38 (55.1)	38 (55.1)		7 (10.1)
20	Di Giambenedetto et al. [45]	2020	Italy	CR	Supplementary Table S3	1	1	75.0		Male, 1 (100)		Severe, 1 (100)	1 (100)	1 (100)	
21	D’Ettorre et al. [46]	2020	Italy	CR	Supplementary Table S3	1	1	52.0		Female, 1 (100)	Ethiopian, 1	Severe, 1 (100)	1 (100)		
22	Elhadi et al. [47]	2020	Libya	CR	Supplementary Table S3	1	1	86.0		Female, 1 (100)			1 (100)	1 (100)	1 (100)
23	Etienne et al. [48]	2020	France	PC	Poor	54	54	54.0	25.2	Male,33 (61.1); Female, 21 (38.9)	African, 26; South American, 3; European, 25	Moderate, 35 (64.8); severe, 14 (25.9); critical, 5 (9.3)			1 (1.9)
24	Faranacci et al. [49]	2020	Italy	CR	Supplementary Table S3	1	1	59.0		Male, 1 (100)			1 (100)	1 (100)	1 (100)
25	Gadelha et al. [50]	2020	Brazil	CS	Supplementary Table S3	2	2	53.0		Male, 2 (100)			2 (100)	1 (50)	
26	Geretti et al. [51]	2020	UK	PC	Good	115	115	55.0		Male, 76 (66.1); Female 39 (33.9)	White, 44; Black, 48; Asian, 1; Other, 13; Unknown, 9				26 (22.6)
27	Gervasoni et al. [52]	2020	Italy	CS	Supplementary Table S3	47	47	51.0		Unknown, 47 (100)			13 (27.7)		2 (4.3)
28	Gudipati et al. [53]	2020	USA	CS	Supplementary Table S3	14	14	44.5		Male, 12 (85.7); Female, 2 (14.3)	African American, 12; Hispanic/ Latino, 2	Severe, 1 (7.1)	8 (57.1)	2 (14.2)	3 (21.4)
29	Guo et al. [54]	2020	China	CS	Supplementary Table S3	14	14	45.3		Male, 13 (92.9); Female 1 (7.1)		Mild, 6 (42.9); Severe, 3 (21.4); Critical, 2 (14.3); Asymptoma-tic, 3 (21.4)	14 (100)	1 (7.1)	2 (14.3)
30	Haddad et al. [55]	2020	USA	CR	Supplementary Table S3	1	1	41.0		Male, 1 (100)			1 (100)	1 (100)	
31	Hadi et al. [56]	2020	USA	CS	Supplementary Table S3	404	404	48.2		Male, 285 (70.5); Female, 119 (29.5)	African America, 201; White, 137; Hispanic/Latino53; Asian, 10; Others, 3		78 (19.3)	27 (6.7)	
32	Harter et al. [57]	2020	Germany	CS	Supplementary Table S3	33	33	47.5		Male, 30 (90.9); Female, 3 (9.1)		Mild, 25 (75.8); Severe, 2 (6.1); Critical, 6 (18.2)	14 (42.4)	6 (18.2)	3 (9.1)
33	Ho et al. [58]	2020	USA	CS	Supplementary Table S3	93	93	58.0	26.7	Male, 67 (72.1); Female, 23 (24.7); Transgender, 3 (3.2)	White, 21; Black 38; Unknown, 34		72 (77.4)	19 (20.4)	19 (20.4)
34	Hu et al. [59]	2020	China	CS	Supplementary Table S3	12	5953	42.4		Male, 10 (83.3); Female, 2 (16.7)		Mild, 9 (75.0); Severe, 2 (16.7); Fatal,1 (8.3)	9 (75)	2 (16.7)	1 (8.3)
35	Huang et al. [60]	2020	China	Cohort	Good	35	6001	52.0		Male, 33 (94.3); Female, 2 (5.7)		Severe, 15 (42.9)			2 (5.7)
36	Inciarte et al. [23]	2020	Spain	PC	Poor	53	5683	44.0		Male, 45 (84.9); Female, 8 (15.1)		Severe, 6 (11.3)	26 (49.1)	4 (7.5)	2 (3.8)
37	Iordanou et al. [61]	2020	Cyprus	CR	Supplementary Table S3	1	1	58.0		Male, 1 (100)	Caucasian, 1	Severe, 1 (100)	1 (100)	1 (100)	
38	Isernia et al. [62]	2020	France	CS	Supplementary Table S3	24	30			Unknown, 24 (100)					2 (8.3)
39	Karmen et al. [63]	2020	USA	RC	Poor	21	21			Unknown, 21 (100)			21 (100)	6 (28.6)	6 (28.6)
40	Khaba et al. [64]	2020	South Africa	CR	Supplementary Table S3	1	1 †	19.0	18.1	Male, 1 (100)	African, 1		1 (100)	1 (100)	1 (100)
41	Kim et al. [65]	2020	South Korea	CR	Supplementary Table S3	1	1	29.0	24.2	Male, 1 (100)	Korean, 1				
42	Kumar et al. [66]	2020	USA	CR	Supplementary Table S3	1	1	50.0		Male, 1 (100)	African American, 1				
43	Li et al. [67]	2020	China	CS	Supplementary Table S3	2	2	54.8		Male, 2 (100)			2 (100)		
44	Liu et al. [68]	2020	China	CS	Supplementary Table S3	20	20	46.5		Male, 5 (25); Female, 15 (75)		Severe, 3 (15)	20 (100)		1 (5)
45	Madge et al. [69]	2020	UK	CS	Supplementary Table S3	18	18	63.0		Male, 14 (77.8); Female, 4 (22.2)	Black, Asian and Minority, 9; Unknown, 9		18 (100)	2 (11.1)	3 (16.7)
46	Maggiolo et al. [24]	2020	Italy	PC	Good	55	2898	54.0		Male, 44 (80); Female, 11 (20)			15 (27.3)		4 (7.3)
47	Mang et al. [70]	2020	Germany	CR	Supplementary Table S3	1	1	52.0		Male, 1 (100)			1 (100)	1 (100)	
48	Marimuthu et al. [71]	2020	India	CS	Supplementary Table S3	6	6	39.9		Male, 3 (50); Female, 2 (33.3), Transgender, 1 (16.7)					
49	Meyerowitz et al. [72]	2020	USA	CS	Supplementary Table S3	36	36	48.1		Male, 21 (58.3); Female, 15 (41.7)	African American, 16; Hispanic, 12; Others, 8	Severe, 8 (22.2); Critical, 7 (19.4)	21 (58.3)	5 (13.9)	2 (5.6)
50	Miyashita and Kuno [73]	2020	USA	CS	Supplementary Table S3	161	161			Male, 125 (77.6); Female, 36 (22.4)			161 (100)	36 (22.4)	23 (14.3)
51	Modi et al. [74]	2020	USA	CR	Supplementary Table S3	1	1	32.0		Unknown, 1 (100)	African American, 1	Mild, 1 (100)	1 (100)		
52	Molina et al. [75]	2020	Spain	RC	Poor	8	902	45.1		Male, 7 (87.5); Female, 1 (12.5)		Mild, 5 (62.5); Severe, 3 (37.5)			1 (12.5)
53	Mondi et al. [76]	2020	Italy	CS	Supplementary Table S3	5	5	46.2		Male, 4 (80); Transgender, 1 (20)		Mild, 3 (60); Severe, 1 (20); Asymptoma-tic 1 (20)			
54	Nakamoto et al. [77]	2020	Japan	CR	Supplementary Table S3	1	1	28.0		Male, 1 (100)			1 (100)		
55	Okoh et al. [78]	2020	USA	CS	Supplementary Table S3	27	27	58.0	31.5	Male, 15 (55.6); Female, 12 (44.4)	African American, 25; Hispanic, 2		13 (48.1)	3 (11.1)	2 (7.4)
56	Parker et al., a [79]	2020	South Africa	CR	Supplementary Table S3	1	1	41.0		Male, 1 (100)			1 (100)	1 (100)	
57	Parker et al., b [80]	2020	South Africa	CS	Supplementary Table S3	24	24	46.2		Male, 6 (25); Female, 18 (75)			19 (79.2)	5 (20.8)	6 (25)
58	Patel and Pella [81]	2020	USA	CR	Supplementary Table S3	1	1	58.0		Male, 1 (100)			1 (100)		
59	Przydzial et al. [82]	2020	USA	CS	Supplementary Table S3	2	2	46.5		Male, 2 (100)			2 (100)		
60	Qasim et al. [83]	2020	USA	CR	Supplementary Table S3	1	1	37.0		Male, 1 (100)		Mild, 1 (100)	1 (100)		
61	Ridgway et al. [84]	2020	USA	CS	Supplementary Table S3	5	5	56.1		Male, 1 (20); Female, 4 (80)		Mild, 5 (100)	5 (100)		
62	Riva et al. [85]	2020	Italy	CS	Supplementary Table S3	3	3	54.9		Male, 2 (66.7); Female, 1 (33.3)			3 (100)	1 (33.3)	
63	Rivas et al. [86]	2020	Panama	CS	Supplementary Table S3	2	2	54.9		Male, 2 (100)			2 (100)	1 (50)	
64	Ruan et al. [87]	2020	China	CS	Supplementary Table S3	4	4	55.8		Male, 4 (100)		Moderate, 2 (50); Severe, 2 (50)	4 (100)		
65	Sasset et al. [88]	2020	Italy	CS	Supplementary Table S3	2	2	54.1		Male, 2 (100)		Severe, 2 (100)	2 (100)	2 (100)	
66	Shalev et al. [89]	2020	USA	CS	Supplementary Table S3	31	31	60.7		Male, 24; Female, 7	Black, 16; White, 5; Hispanic, 9; unknown, 1	Mild, 1 (3.2); Moderate, 2 (6.5); Severe, 21 (67.7); Critical, 7 (22.6)			8 (25.8)
67	Shekhar et al. [90]	2020	USA	CS	Supplementary Table S3	5	5	47.4		Male, 4 (80); Female, 1 (20)			3 (60)	1 (20)	
68	Sigel et al. [91]	2020	USA	CS	Supplementary Table S3	88	88	61.0		Male, 66 (75); Female, 22 (25)	White, 17; Black, 35; Hispanic, 26; Other, 10	Mild, 16 (18.2); Moderate, 54 (61.4); Severe, 18 (20.5)	88 (100)		18 (20.5)
69	Stoeckle et al. [92]	2020	USA	RC	Good	30	30	60.5	27.2	Male, 24 (80); Female 6 (20)	White, 8; Black, 9; Other, 6; Not specified, 7		30 (100)	4 (13.3)	2 (6.7)
70	Su et al. [93]	2020	China	CR	Supplementary Table S3	1	1	32.0		Male, 1 (100)					
71	Sun et al. [94]	2020	Singapore	CR	Supplementary Table S3	1	1	37.0		Male, 1 (100)		Mild, 1 (100)	1 (100)		
72	Suwanwongse and Shabarek, a [95]	2020	USA	CS	Supplementary Table S3	9	9	46.6		Male, 7 (77.8); Female, 2 (22.2)			9 (100)	6 (66.7)	7 (77.8)
73	Suwanwongse and Shabarek, b [96]	2020	USA	CS	Supplementary Table S3	5	5	47.1		Male, 4 (80); Female, 1 (20)			5 (1000)	3 (60)	1 (20)
74	Toombs et al. [97]	2020	UK	CS	Supplementary Table S3	3	3	46.5		Male, 2 (66.7); Female, 1 (33.3)			3 (100)	1 (33.3)	1 (33.3)
75	Vizcarra et al. [25]	2020	Spain	PC	Good	35	2873	53.6	25.5	Male, 30 (85.7); Female, 5 (14.3)			35 (100)	6 (17.1)	
76	Wang et al. [98]	2020	China	CR	Supplementary Table S3	1	1	37.0		Unknown, 1 (100)			1 (100)		
77	Wu et al., a [99]	2020	China	CR	Supplementary Table S3	1	1	49.0		Female, 1 (100)		Moderate, 1 (100)	1 (100)		
78	Wu et al., b [100]	2020	China	CS	Supplementary Table S3	2	2	49.5		Male, 2 (100)			2 (100)		
79	Yamamoto et al. [101]	2020	Japan	CS	Supplementary Table S3	5	5	49.5		Male, 3 (60); Transgender women, 2 (40)		Mild, 5 (100)	5 (100)		
80	Zhang et al. [102]	2020	China	CS	Supplementary Table S3	2	2	49.5		Male, 2 (100)			2 (100)		
81	Zhao et al. [103]	2020	China	CR	Supplementary Table S3	1	1	38.0		Male, 1 (100)			1 (100)		
82	Zhu et al. [104]	2020	China	CR	Supplementary Table S3	1	1	61.0		Male, 1 (100)			1 (100)		

Note: CR = Case report; CS = Case series; HIV + COVID-19 = People living with human immunodeficiency virus(PLHIV) co-infected with COVID-19; PC = Prospective cohort; RC = Retrospective cohort; † Autopsy case.

**Table 2:** Pooled prevalence and 95% confidence interval (CI) of COVID-19 symptoms.

Category	Symptoms	No. of Studies	No. of Patients	Total Number of PLHIV Co-Infected with COVID-19	Pooled Prevalence	95% CI	I^2^	*p*-Value	Supplementary
	Asymptomatic	5	26	196	13.2	3.6, 22.7	75.9	0.002	Figure S1
Constitutional symptoms	Fever	14	614	863	71.1	65.8, 76.4	61.8	0.001	Figure S2
	Lethargy	7	245	600	33.6	20.6, 46.7	90.9	<0.001	Figure S3
Respiratory symptoms	Dry cough	13	571	832	66.3	58.0, 74.7	84.3	<0.001	Figure S4
	Dyspnoea	12	405	799	46.0	34.3, 57.6	91.1	<0.001	Figure S5
	Nasal congestion	5	61	560	8.8	4.7, 12.8	61.2	0.036	Figure S6
	Sore throat	7	109	618	16.0	11.5, 20.5	48.9	0.068	Figure S7
Gastrointestinal manifestations	Abdominal pain	4	18	189	8.4	4.4, 12.3	<0.000	0.455	Figure S8
	Diarrhoea	10	157	739	19.0	14.0, 24.0	62.3	0.005	Figure S9
	Nausea	6	104	555	13.8	6.8, 20.8	80.2	<0.001	Figure S10
Neurological symptoms	Ageusia	5	51	442	9.7	4.4, 15.0	65.2	0.022	Figure S11
	Anosmia	9	81	645	11.5	6.0, 17.0	82.7	<0.001	Figure S12
	Headache	10	158	752	17.9	11.0, 24.8	84.5	<0.001	Figure S13
	Mental status changes/Confusion	3	34	415	8.0	5.4, 10.6	0.0	0.631	Figure S14
Musculoskeletal	Myalgia	11	259	757	28.1	18.7, 37.5	87.2	<0.001	Figure S15
Cardiac symptoms	Chest pain/chest tightness	4	86	455	18.5	14.9, 22.0	0	0.406	Figure S16

**Table 3:** Blood parameters among PLHIV co-infected with COVID-19.

Category of Marker	Blood Parameters [Reference Limits]	Overall Range of Mean	Mean: Decreased, Normal or Elevated	No. of Studies	No. of Patients	Range of Mean in Each Sub-Category
Inflammation	C-reactive protein (mg/dL) [0.3–1.0]	0.06–130	Low	1	20	0.06
			Normal	3	5	0.46–0.5
			Elevated	42	1015	1.13–130
	Ferritin (ng/mL) [M: 20–250; F: 10–125]	0.484–23,647	Normal	6	30	0.484–250.3
			Elevated	19	720	306.7–23,647
	Fibrinogen (mg/dL) [200–400]	437–850	Normal	Nil	Nil	Nil
			Elevated	6	109	437–850
	Interleukin-6 (pg/mL]) [0–16.4 pg/mL; Risk Cut off: 80]	5–139.8	Normal	4	40	5–12.2
			Elevated	9	237	24.7–139.8
	Procalcitonin (ng/mL) [<0.15 to 0.2 mild elevated] >2 elevated; >5 to 10 severe	0.03–162	Normal or Mildly elevated	15	285	0.03–0.97
			Elevated	3	34	2.2–162
	Troponin (ng/mL) [0–4]	0.02–25.3	Normal	2	22	0.02–0.03
			Elevated	2	56	0.89–25.3
Coagulation/fibrinolysis Severity/prognosis (COVID)	D-dimer (ng/mL)	0.4–8854	Normal	4	53	0.4–207
			Elevated	23	444	333–8854
Tissue damage	Lactate dehydrogenase (U/L) [140–280]	169–1200	Normal	8	128	169–273
			Elevated	21	650	312–1200
	AST (U/L) [M: <50; F: <45]	24–477	Normal	10	514	24–48.4
			Elevated	6	107	50.5–477
Liver function	Albumin (g/L) [35–55]	0.03–37.7	Decreased	4	30	0.03–33.2
			Normal	2	22	35.7–37.7
	ALP (U/L) [40–140]	42–198	Normal	7	104	42–128.4
			Elevated	1	1	198
	ALT (U/L) [M: <33; F: <25]	11–363	Normal	10	550	11–32
			Elevated	9	591	37.2–363
	AST (U/L) [M: <50; F: <45]	24–477	Normal	10	514	24–48.4
			Elevated	6	107	50.5–477
Haematological status	Haemoglobin (g/dL) [M: 130–170; F: 120–160]	3.6–16.1	Decreased	4	27	3.6–11.7
			Normal	12	212	12.2–16.1
	Platelets (×10^9^/L) [150–450]	0.197–276,000	Decreased	8	153	0.197–130
			Normal	15	149	148–278
			Elevated	2	3	170 K–276 K
	Leukocyte (×10^9^/L) [4.0–11]	0.46–15.1	Decreased	9	34	0.46–3.94
			Normal	31	880	4.1–10.8
			Elevated	2	6	12.9–15.1
	Lymphocytes (×10^9^/L) [1.5–4.5]	0.4–26	Decreased	27	445	0.4–1.44
			Normal	10	116	1.48–2.45
			Elevated	3	341	13.1–26
	Neutrophil (×10^9^/L) [2.0–7.5]	1.97–10.3	Normal	7	143	1.97–6.7
			Elevated	1	1	10.3

**Table 4:** Subgroup analysis on characteristics of PLHIV co-infected with COVID-19 adherence to Highly active antiretroviral therapy (HAART).

Factors	Characteristics	On HAART (*n* = 19)	Not on HAART (*n* = 9)
HIV profile, mean (range)	Average CD4+	556.9 (range = 201–1827)	331.8 (range = 10–504)
	Average Viral load	24.7 (range = 20–40 RNA copies/mL)	50,492.5 (range = 20–293,313 RNA copies/mL)
Symptoms (Number of individuals with symptom)	General	Nausea (4), decreased appetite (1), malaise (1), lethargy (2) Fever (15), chills (1), myalgia (3), headache (3)	Nausea (1), myalgia (2), Fever (5), chills (2), night sweat (1), headache (3)
	Upper respiratory tract	Sore throat (2), nasal congestion (1)	Sore throat (1)
	Lower respiratory system	Dyspnoea (10/19 = 52.6%), productive cough (4), dry cough (11), tachypnoea (1)	Dyspnoea (6/9 = 66.7%), productive cough (1), dry cough (5)
	Gastrointestinal tract	Abdominal pain (2), diarrhoea (6)	Abdominal pain (2)
	Central nervous system	Mental status changed (1)	Nil
	Cardiovascular system	Palpitation (3)	Chest pain (1), palpitation (1)
Laboratory markers, mean (range)	C-reactive protein (mg/dL)	18.9 (range = 0.49–120); Elevated = 93.8% (Based on 16 pts)	20.9 (range = 0.25–53.2); Elevated = 87.5% (Based on 8 pts)
	D-dimer (ng/mL)	1343.9 (range = 177–6077); Elevated = 83.3% (Based on 12 pts)	433.5 (range = 37–790); Elevated = 75% (Based on 4 pts)
	Ferritin (ng/mL)	880.8 (range = 46–5937); Elevated = 66.7% (Based on 12 pts)	371.5 (range = 0.5–1010); Elevated = 80.0% (Based on 5 pts)
	Fibrinogen (mg/dL)	455.8 (range = 182–624); Elevated = 75% (Based on 4 pts)	450.5 (range = 437–464); Elevated = 100% (Based on 2 pts)
	Interleukin-6 (pg/mL)	60.2 (range = 9.9–213); Elevated = 57.1% (Based on 7 pts)	163.5 (range = 75.9–251); Elevated = 100% (Based on 2 pts)
	Lactate dehydrogenase (U/L)	303.4 (range = 162–467); Elevated = 60% (Based on 5 pts)	616.3 (range = 308–1200); Elevated = 100% (Based on 4 pts)
Chest Imaging Studies	Abnormal findings	18 (94.7)	6 (66.7)
Disease severity	Hospitalization	19 (100)	9 (100)
	ICU admission	10 (52.6)	4 (44.4)
	Mortality	6 (31.6)	3 (33.3)

## Supplementary Materials

The following are available online at https://www.mdpi.com/article/10.3390/ijerph18073554/s1, Table S1: PRISMA checklist, Table S2: Search terms used for final search on 20 September 2020, Table S3: Quality appraisal checklist for case series and case report, Table S4: COVID-19 Disease Severity as defined by World Health Organization, Table S5: Information on comorbidities and lifestyle-related disorders among patients with HIV+COVID-19 in the study, Table S6: HIV profile of patients with HIV+Covid-19, Table S7: Laboratory results, chest imaging findings and vital signs among HIV+Covid-19, Table S8: Pharmacological treatment and supportive care given to HIV+COVID19 patients, Table S9: Findings from studies regarding symptoms experienced by patients throughout clinical course, Table S10: Summary of estimate and its 95% CI for Pooled Incidence proportion of COVID-19 among PLHIV based on data accumulated from cohort studies using leave-one-out Meta-analysis forest plot. Figure S1: Forest plot for pooled prevalence of asymptomatic PLHIV with COVID-19, Figure S2: orest plot for pooled prevalence of fever in PLHIV with COVID-19, Figure S3: Forest plot for pooled prevalence of lethargy in PLHIV with COVID-19, Figure S4: Forest plot for pooled prevalence of dry cough in PLHIV with COVID-19, Figure S5: Forest plot for pooled prevalence of dyspnoea in PLHIV with COVID-19, Figure S6: Forest plot for pooled prevalence of nasal congestion in PLHIV with COVID-19, Figure S7: Forest plot for pooled prevalence of sore throat in PLHIV with COVID-19, Figure S8: Forest plot for pooled prevalence of abdominal pain in PLHIV with COVID-19, Figure S9: Forest plot for pooled prevalence of diarrhoea in PLHIV with COVID-19, Figure S10: Forest plot for pooled prevalence of nausea in PLHIV with COVID-19, Figure S11: Forest plot for pooled prevalence of ageusia in PLHIV with COVID-19, Figure S12: Forest plot for pooled prevalence of anosmia in PLHIV with COVID-19, Figure S13: Forest plot for pooled prevalence of headache in PLHIV with COVID-19, Figure S14: Forest plot for pooled prevalence of altered mental status/confusion in PLHIV with COVID-19, Figure S15: Forest plot for pooled prevalence of myalgia in PLHIV with COVID-19, Figure S16: Forest plot for pooled prevalence of chest pain/chest tightness in PLHIV with COVID-19, Figure S17: Leave-one-out Meta-analysis forest plot for pooled incidence proportion of COVID-19 among PLHIV based on data accumulated from cohort studies.

## Abbreviations

ART	Antiretrovirus therapy
HAART	Highly active antiretroviral therapy
PIP	Pooled incidence proportion
PLHIV	People living with human immunodeficiency virus
