# Supplementary material for: COVID-19 in People Living with HIV: A Systematic Review and Meta-Analysis
Source: Int J Environ Res Public Health. 2021 Mar 30;18(7):3554. doi: 10.3390/ijerph18073554 (PMC8037457; doi:10.3390/ijerph18073554)

Table S1: PRISMA checklist

Section/topic	#	Checklist item	Reported on page #
**TITLE**			
Title	1	Identify the report as a systematic review, meta-analysis, or both.	1
**ABSTRACT**			
Structured summary	2	Provide a structured summary including, as applicable: background; objectives; data sources; study eligibility criteria, participants, and interventions; study appraisal and synthesis methods; results; limitations; conclusions and implications of key findings; systematic review registration number.	2
**INTRODUCTION**			
Rationale	3	Describe the rationale for the review in the context of what is already known.	3
Objectives	4	Provide an explicit statement of questions being addressed with reference to participants, interventions, comparisons, outcomes, and study design (PICOS).	3
**METHODS**			
Protocol and registration	5	Indicate if a review protocol exists, if and where it can be accessed (e.g., Web address), and, if available, provide registration information including registration number.	4
Eligibility criteria	6	Specify study characteristics (e.g., PICOS, length of follow-up) and report characteristics (e.g., years considered, language, publication status) used as criteria for eligibility, giving rationale.	4
Information sources	7	Describe all information sources (e.g., databases with dates of coverage, contact with study authors to identify additional studies) in the search and date last searched.	4
Search	8	Present full electronic search strategy for at least one database, including any limits used, such that it could be repeated.	4
Study selection	9	State the process for selecting studies (i.e., screening, eligibility, included in systematic review, and, if applicable, included in the meta-analysis).	4
Data collection process	10	Describe method of data extraction from reports (e.g., piloted forms, independently, in duplicate) and any processes for obtaining and confirming data from investigators.	4
Data items	11	List and define all variables for which data were sought (e.g., PICOS, funding sources) and any assumptions and simplifications made.	4
Risk of bias in individual studies	12	Describe methods used for assessing risk of bias of individual studies (including specification of whether this was done at the study or outcome level), and how this information is to be used in any data synthesis.	4
Summary measures	13	State the principal summary measures (e.g., risk ratio, difference in means).	4
Synthesis of results	14	Describe the methods of handling data and combining results of studies, if done, including measures of consistency (e.g., I^2) for each meta-analysis.	5
Risk of bias across studies	15	Specify any assessment of risk of bias that may affect the cumulative evidence (e.g., publication bias, selective reporting within studies).	5
Additional analyses	16	Describe methods of additional analyses (e.g., sensitivity or subgroup analyses, meta-regression), if done, indicating which were pre-specified.	N/A

RESULTS			
Study selection	17	Give numbers of studies screened, assessed for eligibility, and included in the review, with reasons for exclusions at each stage, ideally with a flow diagram.	4
Study characteristics	18	For each study, present characteristics for which data were extracted (e.g., study size, PICOS, follow-up period) and provide the citations.	4
Risk of bias within studies	19	Present data on risk of bias of each study and, if available, any outcome level assessment (see item 12).	Appendix 3
Results of individual studies	20	For all outcomes considered (benefits or harms), present, for each study: (a) simple summary data for each intervention group (b) effect estimates and confidence intervals, ideally with a forest plot.	Table 1
Synthesis of results	21	Present results of each meta-analysis done, including confidence intervals and measures of consistency.	11-18
Risk of bias across studies	22	Present results of any assessment of risk of bias across studies (see Item 15).	Table 1
Additional analysis	23	Give results of additional analyses, if done (e.g., sensitivity or subgroup analyses, meta-regression [see Item 16]).	N/A
**DISCUSSION**			
Summary of evidence	24	Summarize the main findings including the strength of evidence for each main outcome; consider their relevance to key groups (e.g., healthcare providers, users, and policy makers).	19-21
Limitations	25	Discuss limitations at study and outcome level (e.g., risk of bias), and at review-level (e.g., incomplete retrieval of identified research, reporting bias).	21
Conclusions	26	Provide a general interpretation of the results in the context of other evidence, and implications for future research.	21
**FUNDING**			
Funding	27	Describe sources of funding for the systematic review and other support (e.g., supply of data); role of funders for the systematic review.	22

Note: Moher D, Liberati A, Tetzlaff J, Altman DG, The PRISMA Group (2009). Preferred Reporting Items for Systematic Reviews and Meta-Analyses: The PRISMA Statement. PLoS Med 6(7): e1000097. doi:10.1371/journal.pmed1000097

Table S2: Search terms used for final search on 20 September 2020				
Searches	Search terms	Medline	Cinahl	PubMed
#1	HIV OR human immunodeficiency virus* OR AIDS OR acquired immunodeficiency syndrome<br><br>*Limiter ENGLISH, HUMAN	289, 055	55, 516	313,724
#2	COVID-19 OR coronavirus OR 2019-NCOV OR nCoV* OR COVID* OR SARS-CoV*<br><br>*Limiter ENGLISH, HUMAN	25, 837	1, 710	28, 197
#3	randomized controlled trials OR controlled clinical trials	193, 021	62, 119	58, 248
#4	review OR meta-analysis	1, 217, 512	195, 123	1, 166, 974
#5	(#1 AND #2)	337	13	385
#6	(#3 OR #4)	1, 349, 546	235, 789	1, 186, 700
#7	(#1 AND #2) NOT (#3 OR #4)	276	11	332

Table S3:: Quality appraisal checklist for case series and case report

Study design	Author	Study Objective	Study design		Study population				Intervention		Outcome measure				Statistical analysis						Compe-ting interests and sources of support
		1	2	3	4	5	6	7	8	9	10	11	12	13	14	15	16	17	18	19	20
		Was the hypothesis/aim/objective of the study clearly stated? (Yes/Partial/No)	Was the study conducted prospectively? (Yes/Unclear/No)	Were the cases collected in more than one centre? (Yes/Unclear/No)	Were patients recruited consecutively? (Yes/Unclear/No)	Were the characteristics of the patients included in the study described? (Yes/Partial/No)	Were the eligibility criteria for entry into the study clearly stated? (Yes/Partial/No)	Did patients enter the study at a similar point in the disease? (Yes/Unclear/No)	Was the intervention of interest clearly described? (Yes/Partial/No)	Were additional interventions (Co-interventions) clearly described? (Yes/Partial/No)	Were relevant outcome measures established a priori ? (Yes/Partial/No)	Were outcome assessors blinded to the interventional that patients received? (Yes/Unclear/No)	Were the relevant outcomes measured using appropriate objective/subjective methods? (Yes/Partial/No)	Were the relevant outcome measures made before and after the intervention? (Yes/Unclear/No)	Were the statistical tests used to assess the relevant outcomes appropriate? (Yes/Unclear/No)	Was follow-up long enough for important events and outcomes to occurs? (Yes/Unclear/No)	Were losses to follow-up reported? (Yes/Unclear/No)	Did the study provide estimates of random variability in the data analysis of relevant outcomes? (Yes/Partial/No)	Were the adverse events reported? (Yes/Partial/No)	Were the conclusions of the study supported by results? (Yes/Unclear/No)	Were both competing interests and sources of support for the study reported? (Yes/Partial/No)
Case report	Baluku et al.,	*Yes*	*Yes*	*No*	*Unclear*	*Partial*	*No*	*Unclear*	*Yes*	*Yes*	*Partial*	*Unclear*	*Yes*	*No*	*No*	*Unclear*	*Unclear*	*No*	*Yes*	*Yes*	*Partial*
Case report	*Chen et al.,*	*Yes*	*Yes*	*No*	*Unclear*	*Partial*	*No*	*Unclear*	*Yes*	*Yes*	*Partial*	*Unclear*	*Yes*	*No*	*No*	*Unclear*	*Unclear*	*No*	*Yes*	*Yes*	*Yes*
Case report	Chiappe et al.,	*Yes*	*Yes*	*No*	*Unclear*	*Partial*	*No*	*Unclear*	*Yes*	*Yes*	*Partial*	*Unclear*	*Yes*	*No*	*No*	*Unclear*	*Unclear*	*No*	*Yes*	*Yes*	*Yes*
Case report	Cipolat and Sprinz	*Yes*	*Yes*	*No*	*Unclear*	*Partial*	*No*	*Unclear*	*Yes*	*Yes*	*Partial*	*Unclear*	*Yes*	*No*	*No*	*Unclear*	*Unclear*	*No*	*Yes*	*Yes*	*No*
Case report	*Coleman et al.,*	*Yes*	*Yes*	*No*	*Unclear*	*Partial*	*No*	*Unclear*	*Yes*	*Yes*	*Partial*	*Unclear*	*Yes*	*No*	*No*	*Unclear*	*Unclear*	*No*	*Yes*	*Yes*	*Yes*
Case Report	D'Ettorre et al.,	*Yes*	*Yes*	*No*	*Unclear*	*Partial*	*No*	*Unclear*	*Yes*	*Yes*	*Partial*	*Unclear*	*Yes*	*No*	*Yes*	*Unclear*	*Unclear*	*Partial*	*Yes*	*Yes*	*Yes*
Case Report	Di Giambenedetto	*Yes*	*Yes*	*No*	*Unclear*	*Partial*	*No*	*Unclear*	*Yes*	*Yes*	*Partial*	*Unclear*	*Yes*	*No*	*No*	*Unclear*	*Unclear*	*No*	*Yes*	*Yes*	*Yes*

	et al.,																		
Case report	Elhadi et al.,	Yes	Yes	No	Unclear	Partial	No	Unclear	Yes	Yes	Partial	Unclear	Yes	No	No	Unclear	Unclear	No	Yes	Yes	Yes
Case Report	Faranacci et al.,	Yes	Yes	No	Unclear	Partial	No	Unclear	Yes	Yes	Partial	Unclear	Yes	No	No	Unclear	Unclear	No	Yes	Yes	No
Case report	Haddad et al.,	Yes	Yes	No	Unclear	Partial	No	Unclear	Yes	Yes	Partial	Unclear	Yes	No	No	Unclear	Unclear	No	Yes	Yes	Yes
Case report	Iordanou et al.,	Yes	Yes	No	Unclear	Partial	No	Unclear	Yes	Yes	Partial	Unclear	Yes	No	Unclear	Unclear	Unclear	Partial	Yes	Yes	Partial
Case report	Khaba et al.,	Yes	Yes	No	Unclear	Yes	No	Unclear	Yes	Yes	Partial	Unclear	Yes	No	No	Unclear	Unclear	No	Yes	Yes	Yes
Case report	Kim et al.,	Yes	Yes	No	Unclear	Yes	No	Unclear	Yes	Yes	Partial	Unclear	Yes	No	Unclear	Unclear	Unclear	Partial	Partial	Yes	Partial
Case report	Kumar et al.,	Yes	Yes	No	Unclear	Partial	No	Unclear	Yes	Yes	Partial	Unclear	Yes	No	No	Unclear	Unclear	No	Partial	Yes	No
Case report	Mang et al.,	Yes	Yes	No	Unclear	Partial	No	Unclear	Yes	Yes	Partial	Unclear	Yes	No	No	Unclear	Unclear	No	Yes	Yes	Yes
Case report	Modi et al,.	Yes	Yes	No	Unclear	Partial	No	Unclear	Yes	Yes	Partial	Unclear	Yes	No	Unclear	Unclear	Unclear	Partial	Yes	Yes	Partial
Case report	Nakamoto et al.,	Yes	Yes	No	Unclear	Partial	No	Unclear	Yes	Yes	Partial	Unclear	Yes	No	No	Unclear	Unclear	No	Yes	Yes	No
Case report	Parker et al., a	Yes	Yes	No	Unclear	Partial	No	Unclear	Yes	Yes	Partial	Unclear	Yes	No	No	Unclear	Unclear	No	Yes	Yes	Yes
Case report	Patel and Pella	Yes	Yes	No	Unclear	Partial	No	Unclear	Yes	Yes	Partial	Unclear	Yes	No	Unclear	Unclear	Unclear	Partial	Yes	Yes	Yes
Case report	Qasim et al.	Yes	Yes	No	Unclear	Partial	No	Unclear	Yes	Yes	Partial	Unclear	Yes	No	Unclear	Unclear	Unclear	Partial	Yes	Yes	No
Case report	Su et al.,	Yes	Yes	No	Unclear	Partial	No	Unclear	Yes	Yes	Partial	Unclear	Yes	No	Unclear	Unclear	Unclear	Partial	Partial	Yes	Yes
Case report	Sun et al.,	Yes	Yes	No	Unclear	Partial	No	Unclear	Yes	Yes	Partial	Unclear	Yes	No	Unclear	Unclear	Unclear	Partial	Yes	Yes	No
Case report	Wang et al.,	Yes	Yes	No	Unclear	Partial	No	Unclear	Yes	Yes	Partial	Unclear	Yes	No	Unclear	Unclear	Unclear	Partial	Yes	Yes	Yes
Case report	Wu et al., a	Yes	Yes	No	Unclear	Partial	No	Unclear	Yes	Yes	Partial	Unclear	Yes	No	Unclear	Unclear	Unclear	Partial	Yes	Yes	Yes
Case report	Zhao et al.,	Yes	Yes	No	Unclear	Partial	No	Unclear	Yes	Yes	Partial	Unclear	Yes	No	Unclear	Unclear	Unclear	Partial	Yes	Yes	Yes
Case report	Zhu et al.,	Yes	Yes	No	Unclear	Partial	No	Unclear	Yes	Yes	Partial	Unclear	Yes	No	No	Unclear	Unclear	No	Yes	Yes	Yes
Case series	*Adachi et al.,*	Yes	Yes	Unclear	Unclear	Partial	No	Unclear	Yes	Yes	Partial	Unclear	Yes	No	No	Unclear	Unclear	No	Yes	Yes	Partial
Case series	Altuntas et al.,	Yes	Yes	No	Unclear	Partial	No	Unclear	Yes	Yes	Partial	Unclear	Yes	No	No	Unclear	Unclear	No	Yes	Yes	No
Case series	Benkovic et al.,	Yes	Yes	No	Unclear	Partial	No	Unclear	Yes	Yes	Partial	Unclear	Yes	No	No	Unclear	Unclear	No	Yes	Yes	Yes
Case series	*Byrd et al.,*	Yes	Yes	No	Unclear	Partial	No	Unclear	Yes	Yes	Partial	Unclear	Yes	No	No	Unclear	Unclear	No	Yes	Yes	Yes
Case series	Calza et al., a	Yes	Yes	No	Unclear	Partial	No	Unclear	Yes	Yes	Partial	Unclear	Yes	No	Unclear	Unclear	Unclear	Partial	Yes	Yes	Yes
Case series	*Calza et al., b*	Yes	Yes	No	Unclear	Yes	No	Unclear	Yes	Yes	Partial	Unclear	Yes	No	No	Unclear	Unclear	Yes	No	Yes	Yes
Case series	*Childs et al.,*	Yes	Yes	No	Unclear	Partial	No	Unclear	Yes	Yes	Partial	Unclear	Yes	No	Unclear	Unclear	Unclear	Partial	Yes	Yes	Yes

Case series	*Collins et al.,*	*Yes*	*Yes*	*Yes*	*Unclear*	*Yes*	*No*	*Unclear*	*Yes*	*Yes*	*Partial*	*Unclear*	*Yes*	*No*	*Unclear*	*Unclear*	*Unclear*	*Partial*	*Yes*	*Yes*	*Partial*
Case series	*Dandachi et al.,*	*Yes*	*Yes*	*Yes*	*Yes*	*Yes*	*No*	*Unclear*	*Yes*	*Yes*	*Partial*	*Unclear*	*Yes*	*No*	*Yes*	*Unclear*	*Unclear*	*Partial*	*Yes*	*Yes*	*Yes*
Case series	Di Biagio et al., a	*Yes*	*Yes*	*Unclear*	*Unclear*	*Partial*	*No*	*Unclear*	*Yes*	*Yes*	*Partial*	*Unclear*	*Yes*	*No*	*No*	*Unclear*	*Unclear*	*No*	*Yes*	*Yes*	*Partial*
Case series	Di Biagio et al., b	*Yes*	*Yes*	*Yes*	*Unclear*	*Yes*	*No*	*Unclear*	*Yes*	*Yes*	*Partial*	*Unclear*	*Yes*	*No*	*Yes*	*Unclear*	*Unclear*	*Yes*	*Yes*	*Yes*	*Yes*
Case series	Gadelha et al.,	*Yes*	*Yes*	*No*	*Unclear*	*Partial*	*No*	*Unclear*	*Yes*	*Yes*	*Partial*	*Unclear*	*Yes*	*No*	*No*	*Unclear*	*Unclear*	*No*	*Yes*	*Yes*	*No*
Case series	Gervasoni et al.,	*Yes*	*No*	*No*	*Unsure*	*Yes*	*No*	*Unclear*	*Yes*	*Yes*	*Partial*	*Unclear*	*Yes*	*No*	*No*	*Unclear*	*Unclear*	*Partial*	*Yes*	*Yes*	*Partial*
Case series	Gudipati et al.,	*Yes*	*Yes*	*No*	*Unclear*	*Partial*	*No*	*Unclear*	*Yes*	*Yes*	*Partial*	*Unclear*	*Yes*	*No*	*No*	*Unclear*	*Unclear*	*No*	*Yes*	*Yes*	*Yes*
Case series	Guo et al.,	*Yes*	*Yes*	*Unclear*	*Unclear*	*Partial*	*No*	*Unclear*	*Yes*	*Yes*	*Partial*	*Unclear*	*Yes*	*No*	*No*	*Unclear*	*Unclear*	*No*	*Yes*	*Yes*	*Yes*
Case series	Hadi et al.,	*Yes*	*Yes*	*Yes*	*Unclear*	*Yes*	*No*	*Unclear*	*Yes*	*Yes*	*Partial*	*Unclear*	*Yes*	*No*	*Yes*	*Unclear*	*Unclear*	*Partial*	*Yes*	*Yes*	*Yes*
Case series	Harter et al.,	*Yes*	*Yes*	*Unclear*	*Unclear*	*Partial*	*No*	*Unclear*	*Yes*	*Yes*	*Partial*	*Unclear*	*Yes*	*No*	*No*	*Unclear*	*Unclear*	*No*	*Yes*	*Yes*	*Partial*
Case series	Ho et al.,	*Yes*	*No*	*Yes*	*Yes*	*Yes*	*No*	*Unclear*	*Yes*	*Yes*	*Partial*	*Unclear*	*Yes*	*No*	*Yes*	*Unclear*	*Unclear*	*Partial*	*Yes*	*Yes*	*Yes*
Case series	Hu et al.,	*Yes*	*Yes*	*Yes*	*Unclear*	*Partial*	*No*	*Unclear*	*Yes*	*Yes*	*Partial*	*Unclear*	*Yes*	*No*	*Unclear*	*Unclear*	*Unclear*	*Partial*	*Yes*	*Yes*	*Yes*
Case series	Isernia et al.,	*Yes*	*Yes*	*No*	*Unclear*	*Partial*	*No*	*Unclear*	*Yes*	*Yes*	*Partial*	*Unclear*	*Yes*	*No*	*No*	*Unclear*	*Unclear*	*No*	*Yes*	*Yes*	*Yes*
Case series	Li et al.,	*Yes*	*Yes*	*Unclear*	*Unclear*	*Partial*	*No*	*Unclear*	*Yes*	*Yes*	*Partial*	*Unclear*	*Yes*	*No*	*No*	*Unclear*	*Unclear*	*No*	*Yes*	*Yes*	*Yes*
Case series	Liu et al.,	*Yes*	*No*	*No*	*Unclear*	*Yes*	*No*	*Unclear*	*Yes*	*Yes*	*Partial*	*Unclear*	*Yes*	*No*	*Yes*	*Unclear*	*Unclear*	*Yes*	*Partial*	*Yes*	*Yes*
Case series	Madge et al.,	*Yes*	*No*	*No*	*Unclear*	*Partial*	*No*	*Unclear*	*Yes*	*Yes*	*Partial*	*Unclear*	*Yes*	*No*	*No*	*Unclear*	*Unclear*	*No*	*No*	*Yes*	*Yes*
Case series	Marimuthu et al.,	*Yes*	*Yes*	*Yes*	*Unclear*	*Partial*	*No*	*Unclear*	*Yes*	*Yes*	*Partial*	*Unclear*	*Yes*	*No*	*No*	*Unclear*	*Unclear*	*No*	*Yes*	*Yes*	*Partial*
Case series	Meyerowitz et al.,	*Yes*	*Yes*	*No*	*Unclear*	*Partial*	*No*	*Unclear*	*Yes*	*Yes*	*Partial*	*Unclear*	*Yes*	*No*	*No*	*Unclear*	*Unclear*	*No*	*Yes*	*Yes*	*Yes*
Case series	Miyashita and Kuno	*Yes*	*No*	*Yes*	*No*	*Partial*	*No*	*Unclear*	*Yes*	*Yes*	*Partial*	*Unclear*	*Yes*	*No*	*Yes*	*Unclear*	*Unclear*	*Partial*	*Yes*	*Yes*	*Partial*
Case series	Mondi et al.,	*Yes*	*Yes*	*No*	*Unclear*	*Partial*	*No*	*Unclear*	*Yes*	*Yes*	*Partial*	*Unclear*	*Yes*	*No*	*No*	*Unclear*	*Unclear*	*No*	*Yes*	*Yes*	*Partial*
Case Series	Okoh et al.,	*Yes*	*Yes*	*No*	*Unclear*	*Yes*	*No*	*Unclear*	*Yes*	*Yes*	*Partial*	*Unclear*	*Yes*	*No*	*Unclear*	*Unclear*	*Unclear*	*Partial*	*Yes*	*Yes*	*No*
Case series	Parker et al., b	*Yes*	*Yes*	*No*	*Yes*	*Partial*	*No*	*Unclear*	*Yes*	*Yes*	*Partial*	*Unclear*	*Yes*	*No*	*Yes*	*Unclear*	*Unclear*	*Yes*	*Yes*	*Yes*	*Yes*
Case series	Przydzial et al.,	*Yes*	*Yes*	*No*	*Unclear*	*Partial*	*No*	*Unclear*	*Yes*	*Yes*	*Partial*	*Unclear*	*Yes*	*No*	*Unclear*	*Unclear*	*Unclear*	*Partial*	*Yes*	*Yes*	*No*
Case series	Ridgway et al.,	*Yes*	*Yes*	*No*	*Unclear*	*Partial*	*No*	*Unclear*	*Yes*	*Yes*	*Partial*	*Unclear*	*Yes*	*No*	*No*	*Unclear*	*Unclear*	*No*	*Yes*	*Yes*	*Yes*
Case series	Riva et al.,	*Yes*	*Yes*	*No*	*Unclear*	*Partial*	*No*	*Unclear*	*Yes*	*Yes*	*Partial*	*Unclear*	*Yes*	*No*	*No*	*Unclear*	*Unclear*	*No*	*Yes*	*Yes*	*Partial*
Case series	Rivas et al.,	*Yes*	*Yes*	*No*	*Unclear*	*Partial*	*No*	*Unclear*	*Yes*	*Yes*	*Partial*	*Unclear*	*Yes*	*No*	*No*	*Unclear*	*Unclear*	*No*	*Yes*	*Yes*	*Partial*
Case series	Ruan et al.,	*Yes*	*Yes*	*Unclear*	*Unclear*	*Partial*	*No*	*Unclear*	*Yes*	*Yes*	*Partial*	*Unclear*	*Yes*	*No*	*No*	*Unclear*	*Unclear*	*No*	*Yes*	*Yes*	*Partial*
Case series	Sasset et al.,	*Yes*	*Yes*	*No*	*Unclear*	*Partial*	*No*	*Unclear*	*Yes*	*Yes*	*Partial*	*Unclear*	*Yes*	*No*	*No*	*Unclear*	*Unclear*	*No*	*Yes*	*Yes*	*Yes*
Case series	Shalev et al.,	*Yes*	*No*	*No*	*Unclear*	*Yes*	*No*	*Unclear*	*Yes*	*Yes*	*Partial*	*Unclear*	*Yes*	*No*	*No*	*Unclear*	*Unclear*	*Yes*	*Yes*	*Yes*	*Partial*
Case series	Shekhar et al.,	*Yes*	*Yes*	*No*	*Unclear*	*Partial*	*No*	*Unclear*	*Yes*	*Yes*	*Partial*	*Unclear*	*Yes*	*No*	*No*	*Unclear*	*Unclear*	*No*	*Yes*	*Yes*	*Yes*
Case series	Sigel et al.,	*Yes*	*Yes*	*No*	*Unclear*	*Yes*	*No*	*Unclear*	*Yes*	*Yes*	*Partial*	*Unclear*	*Yes*	*No*	*Yes*	*Unclear*	*Unclear*	*Yes*	*Yes*	*Yes*	*Yes*
Case series	Suwanwongse and Shabarek, a	*Yes*	*Yes*	*No*	*Unclear*	*Partial*	*No*	*Unclear*	*Yes*	*Yes*	*Partial*	*Unclear*	*Yes*	*No*	*No*	*Unclear*	*Unclear*	*No*	*Yes*	*Yes*	*No*
Case series	Suwanwongse and Shabarek, b	*Yes*	*Yes*	*No*	*Unclear*	*Partial*	*No*	*Unclear*	*Yes*	*Yes*	*Partial*	*Unclear*	*Yes*	*No*	*No*	*Unclear*	*Unclear*	*No*	*Yes*	*Yes*	*No*
Case series	Toombs et al.,	*Yes*	*Yes*	*No*	*Unclear*	*Partial*	*No*	*Unclear*	*Yes*	*Yes*	*Partial*	*Unclear*	*Yes*	*No*	*No*	*Unclear*	*Unclear*	*No*	*Yes*	*Yes*	*Yes*
Case series	Wu et al., b	*Yes*	*Yes*	*No*	*Unclear*	*Partial*	*No*	*Unclear*	*Yes*	*Yes*	*Partial*	*Unclear*	*Yes*	*No*	*No*	*Unclear*	*Unclear*	*No*	*Yes*	*Yes*	*Yes*

Case series	Yamamoto et al.,	*Yes*	*Yes*	*No*	*Unclear*	*Partial*	*No*	*Unclear*	*Yes*	*Yes*	*Partial*	*Unclear*	*Yes*	*No*	*No*	*Unclear*	*Unclear*	*No*	*Yes*	*Yes*	*Yes*
Case series	Zhang et al.,	*Yes*	*Yes*	*No*	*Unclear*	*Partial*	*No*	*Unclear*	*Yes*	*Yes*	*Partial*	*Unclear*	*Yes*	*No*	*No*	*Unclear*	*Unclear*	*No*	*Yes*	*Yes*	*Yes*
Case series	*Blanco et al.,*	*Yes*	*Yes*	*No*	*Unclear*	*Partial*	*No*	*Unclear*	*Yes*	*Yes*	*Partial*	*Unclear*	*Yes*	*No*	*No*	*Unclear*	*Unclear*	*No*	*Yes*	*Yes*	*Partial*
Note: This quality appraisal checklist for case series studies was developed by Institute of Health Economics.																					

Table S4: COVID-19 Disease Severity as defined by World Health Organization	
Severity	Description
Mild disease	Symptomatic patients meeting **the case definition*** for COVID-19 without evidence of viral pneumonia or hypoxia.<br><br>*Clinical presentation: Most persons experience symptoms such as fever, cough, fatigue, anorexia shortness of breath or myalgias; Non-specific symptoms could be sore throat, nasal congesting, headache, diarrhoea, nausea and vomiting. Some patients may present with symptoms such as anosmia, ageusia preceding the onset of respiratory symptoms.
Moderate disease (*Pneumonia*)	Adolescent or adults with clinical **signs of pneumonia** (fever, cough, dyspnoea, fast breathing) but no signs of severe pneumonia, including $SpO_2 \geq 90\%$ on room air.<br><br>*Fast breathing, < 2months, ≥ 60 breaths/min; 2-11 months, ≥ 50 breaths/min; 1-5 years, ≥ 40 breaths/min.
Severe disease (*Severe Pneumonia*)	Adolescent or adult with clinical **sign of pneumonia** (fever, cough, dyspnoea, fast breathing) plus one of the following:<br>-Respiratory rate >30 breaths/min;<br>-Severe respiratory distress or $SpO_2 < 90\%$ on room air.<br>While the diagnosis can be made on clinical grounds; chest imaging (radiographs, CT scane ultrasound) may assist in diagnosis and identify or exclude pulmonary complications.
Critical disease (*Acute respiratory distress syndrome, ARDS*)	Onset: Within 1 week of a known clinical insult (Pneumonia) or new or worsening respiratory symptoms<br><br>Chest imaging (Radiographs, CT scan or lung ultrasound): Bilateral opacities, not fully explained by volume overload, lobar or lung collapse or nodules.<br><br>Origin of pulmonary infiltrates: Respiratory failure not fully explained by cardiac failure or fluid overload. Need objective assessment (such as echocardiography) to exclude hydrostatic cause of infiltrates/oedema if no risk factor present.<br><br>Oxygen impairment in adults:<br>Mild ARDS: 200 mmHg < $PaO_2/FiO_2 \leq$ 300 mmHg (with PEEP/CPAP ≥ 5 cmH_20).<br>Moderate ARDS: 100 mmHg < $PaO_2/FiO_2 \leq$ 200 mmHg (with PEEP ≥ 5 cmH_20).<br>Severe ARDS: $PaO_2/FiO_2 \leq$ 100 mmHg (with PEEP ≥ 5 cmH_20).
Critical disease	Adults: Acute life-threatening organ dysfunction caused by a dysregulated

(Sepsis)	host response to suspected or proven infection. Sign of organ dysfunction include altered mental status, difficult or fast breathing, low oxygen saturation, reduced urine output, fast heart rate, weak pulse, cold extremities or low blood pressure, skin mottling, laboratory evidence of coagulopathy, thrombocytopenia, acidosis, high lactate, or hyperbilirubinemia.
Critical disease (Septic shock)	Adults: Persistent hypotension despite volume resuscitation, requiring vasopressor to maintain MAP $\geq$ 65 mmHg and serum lactate level > 2mmol/L.

Table S5. **Information on comorbidities and lifestyle-related disorders among patients with HIV+COVID-19 in the study.**

Author	Other comorbidities or None	Neurological disorders	Neoplastic disorders	Haematological disorders	Psychiatric disorders	Respiratory	CVD	Liver disorders	Kidney disorders	Endocrine and Metabolic Disorders	Communicable disease	Lifestyle disorders
Adachi et al.,												
Altuntas et al.,					Bipolar disorder, 1	COPD, 1	HPT, 1			DM, 1	HBV, 1	
Baluku et al.,	None, 1											
Benkovic et al.,			Lymphoma, 1				HPT, 3; Atrial Fibrillation, 1			DM, 1; HLD, 3		
Blanco et al.,						Asthma, 1	Ischemic heart disease, 1				*Pneumocystis jirovecii* pneumonia, 3	
Boulle et al.,						Asthma, 228	HPT, 740		NS CKD, 103	DM, 430	TB, 188	
Byrd et al.,		Stroke, 1; Dementia, 1,	Malignancy, 1			Asthma, 1, COPD, 1	HPT, 6; Coronary artery disease, 1	Cirrhosis, 1	ESRD, 1	DM, 4; HLD, 1	Histoplasmosis 1	Smoking, 5; Alcohol consumption, 1; Illicit drug use, 1
Calza et al., b												
Calza et al., a						Asthma, 3	HPT, 11			DM, 4		
Chen et al.,												
Chiappe et al.,											*Cryptococccus neoformans* infection, 1	
Childs et al.,							HPT, 6		NS CKD, 5	DM, 4		
Cipolat and Sprinz							HPT, 1					
Coleman et al.,						Asthma, 1					*Pneumocystis jirovecii*	

											pneumonia, 1	
Collins et al.,	Obstructive sleep apnoea, 1		Malignancies, 3		Anxiety disorder, 8; Depression, 8	Asthma, 1; COPD, 3; Pulmonary fibrosis, 1	HPT, 14; NS CVD, 6		NS CKD, 5	DM, 9; Dyslipid-aemia, 12	*Pneumocystis jirovecii* pneumonia, 5; *Streptococcus pneumoniae* bacteraemia/ Pneumococcal pneumonia, 1; HBV, 1; HCV, 3; NS Opportunistic infection, 1	Smoking, 3; Alcohol consumption, 10; Illicit drug use, 10
Dandachi et al.,			Malignancies, 13			Chronic Lung disease, 49	HPT, 133; NS CVD, 30	NS liver disease, 28	NS CKD, 48	DM, 61		Obesity, 83
Del Amo et al.,												
Di Biagio et al., a			Adenocarcinoma, 1				HPT, 3	NS liver disease, 1				Obesity, 1
Di Biagio et al., b							HPT, 31; NS CVD, 9			DM, 11	NS Pneumonia, 32	
Di Giambenedetto et al.,							HPT, 1					
D'Ettorre et al.,											NS Pneumonia, 1	Smoke, 1
Elhadi et al.,										DM, 1	NS Pneumonia, 1	
Etienne et al.,			Malignancies, 2			Asthma, 4; COPD, 4	HPT, 12; Heart disease, 4; NS CVD, 25	Cirrhosis, 1; NS liver disease, 14	NS CKD, 3	DM, 5		Smoke, 7; Obesity, 11
Faranacci et al.,											*Pneumocystis jirovecii* pneumonia, 1	
Gadelha et al.,					Anxiety disorder, 1						HBV, 1; TB, 2	Illicit drug use, 2
Geretti et al.,	Rheumato-logical disease, 6	NS Neuro-logical	Malignancies, 3	Chronic haematological disease, 4		Asthma, 12; Chronic lung disease, 13	Heart disease, 20	NS liver disease, 9	NS CKD, 19	DM, 24; Metabolic	*Myco-bacterium chimera*	Smoke, 11; Obesity, 19

		disorder, 7; Dementia, 3								syndrome5	meningitis, 9	
Gervasoni et al.,	SOT, 1; Gastritis, 2	NS Neuro-logical disorder, 3; Dementia, 2				COPD, 2	HPT, 14; NS CVD, 2		NS CKD, 4	DM, 3; Dyslipid-aemia, 2	HBV, 5; HCV, 5	
Gudipati et al.,						COPD, 3	HPT, 11; Congestive heart failure, 3		NS CKD, 4; ESRD, 2	DM, 6		Smoking, 7; Alcohol consumption, 7; Obesity, 8
Guo et al.,		Stroke, 1	Malignancies, 2	Anaemia, 1		COPD, 1	HPT, 5; Atrial Fibrillation, 1			DM, 1	*Pneumocystis jirovecii* pneumonia, 1; TB, 1	Smoke, 2
Haddad et al.,											Herpes Simplex infection, 1	
Hadi et al.,						Chronic lower respiratory disease, 101	HPT, 187		NS CKD, 67	Dyslipid-aemia, 89	Influenza, 57	Illicit drug use, 56
Harter et al.,						COPD, 6	HPT, 10; NS CVD, 3		NS CKD, 2	DM, 4	HBV, 1	
Ho et al.,	Auto-immune disease, 4; SOT, 5		Malignancies, 8			Chronic lung disease, 25	HPT, 49; Heart disease, 17		NS CKD, 16; ESRD, 7	DM, 32		Smoke, 14
Hu et al.,							HPT, 2		NS CKD, 1		TB, 1	
Huang et al.,												
Inciarte et al.,	Acute tonsillitis, 1; Urinary tract infection, 2	NS Neuro-logical disorder, 6				COPD, 3	HPT, 9; Peripheral artery disease, 13; NS CVD, 2	Fatty liver syndrome, 3	NS CKD, 2	DM, 5	*Streptococcus pneumoniae* bacteraemia/ Pneumococcal pneumonia, 2; Syphilis, 2; TB, 1	Smoke, 16; Alcohol consumption, 24; Illicit drug use, 11

Iordanou et al.,	None, 1											
Isernia et al.,	SOT, 2	Stroke, 5; Dementia, 1	Malignancies, 2			COPD, 1; Pulmonary embolism, 2	HPT, 10; Atrial fibrillation, 1; Heart disease, 4		NS CKD, 3; ESRD, 1	DM, 8; Dyslipid-aemia, 1	Influenza, 1, Herpes simplex infection, 1; TB, 1	Alcohol consumption, 1
Karmen et al.,											*Pneumocystis jirovecii* pneumonia, 6	
Khaba et al.,	None, 1											
Kim et al.,												
Kumar et al.,	SOT, 1					Asthma, 1	HPT, 1	NS liver disease, 1	ESRD, 1			
Li et al.,												
Liu et al.,					NS Psychosis, 1	COPD, 1	HPT, 4	NS liver disease, 3		DM, 1	HBV, 1; HCV, 8; Syphilis, 4	
Madge et al.,		Stroke, 3	Malignancy, 1			COPD, 4	HPT, 7		NS CKD, 1; ESRD, 4	DM, 6		
Maggiolo et al.,	Gastro-enteric, 6	NS Neuro-logical disorder, 4	Malignancies, 5				HPT, 12; NS CVD, 9			DM, 3	HBV, 5; HCV, 1	
Mang et al.,							Peripheral artery disease, 1				*Pneumocystis jirovecii* pneumonia, 1	
Marimuthu et al.,							HPT, 2				Candidiasis, 1	
Meyerowitz et al.,		Stroke, 5; Dementia, 2		Essential thrombocythemia, 1		Asthma, 1; COPD, 3	HPT, 11; Congestive heart failure, 4; Coronary heart disease, 1	NS liver disease, 2	NS CKD, 6; ESRD, 2	DM, 7; HLD, 8		Smoke, 3; Obesity, 3
Miyashita and Kuno							HPT, 74; Heart failure, 15		NS CKD, 39	DM, 46; Dyslipid-aemia, 55		
Modi et al,.	SOT, 1											

Molina et al.,			Stable Hodgkin lymphoma stage IV, 1; stable supraglottic squamous cell carcinoma, 1			Bronchiectasis, 1; COPD, 1	HPT, 2			DM, 2	*Pneumocystis jirovecii* pneumonia, 5; HCV, 4; NS Opportunistic infection 2	
Mondi et al.,		Stroke, 1; NS Neuro-logical disorder, 1				Asthma, 1	Heart disease, 1; Myocardial infarction, 1				NS Pneumonia, 5; HBV, 1	
Nakamoto et al.,											HBV, 1; Syphilis, 1	Smoke, 1; Alcohol consumption, 1
Okoh et al.,							HPT, 16; Coronary heart disease, 1		NS CKD, 10; ESRD, 6	DM, 9		
Parker et al., a											*Pneumocystis jirovecii* pneumonia, 1	
Parker et al., b						Chronic lung disease, 2	HPT, 8; Heart disease, 1		NS CKD, 2	DM, 10; Dyslipid-aemia, 1	TB, 4	Smoke, 8; Obesity, 5
Patel and Pella						Chronic bronchitis, 1	HPT, 1					
Przydzial et al.,							HPT, 1			HLD, 1	Streptococcus pneumoniae bacteraemia/ Pneumococcal pneumonia, 1	Illicit drug use, 1
Qasim et al.					Anxiety disorder, 1; Depression, 1							
Ridgway et al.,		Stroke, 2				COPD, 2; Broncho-	HPT, 2;			DM, 2;		Obesity, 3

						esophageal fistula, 1; Pulmonary embolism, 1	Congestive heart failure, 1			Addison disease, 1; HLD, 2		
Riva et al.,							HPT, 3;				Influenza, 1	HLD, 3
Rivas et al.,											TB, 2	
Ruan et al.,							HPT, 1; Heart disease, 1; Coronary heart disease, 1			DM, 1	*Pneumocystis jirovecii* pneumonia, 1	
Sasset et al.,							HPT, 2; Atrial Fibrillation, 1					Obesity, 2
Shalev et al.,						COPD, 8	HPT 21		NS CKD, 7	DM, 13		Smoke, 13; Obesity, 9
Shekhar et al.,					Depression, 2		Peripheral artery disease, 1		NS CKD, 1	DM, 1; HLD, 1		Alcohol consumption, 1
Sigel et al.,	SOT, 4		Malignancies, 15			COPD, 8	HPT, 33; Coronary heart disease, 6	Cirrhosis, 5	NS CKD, 19	DM, 24		Smoke, 48; Obesity, 9
Stoeckle et al.,						Asthma, 3; COPD, 4	HPT, 12; Congestive heart failure, 1; Coronary heart disease, 1	Cirrhosis, 1	ESRD, 2	DM, 8	HBV, 6; HCV, 1	Smoke, 5
Su et al.,												
Sun et al.,												
Suwanwongse and Shabarek, a						COPD, 4	HPT, 5; Atrial Fibrillation, 2			DM, 3; HLD, 4	Syphilis, 1; HCV, 3	Obesity, 1
Suwanwongse and Shabarek, b	Benign prostatic hyper-					Asthma, 2	HPT, 3		ESRD, 1	DM, 2; HLD, 1	HCV, 2	Alcohol consumption, 1

	trophy, 1											
Toombs et al.,	G6PD, 1; SOT, 1	Stroke, 3					HPT, 2			DM, 2	*Pneumocystis jirovecii* pneumonia, 1; Latent TB, 1	Smoke, 1; Obesity, 1
Vizcarra et al.,			Malignancies, 3			Chronic lung disease 8	HPT, 15; NS CVD, 10	NS liver disease, 16	NS CKD, 3	DM, 6		Smoke, 34
Wang et al.,											Syphilis, 1	
Wu et al., a												
Wu et al., b										DM, 1	*Pneumocystis jirovecii* pneumonia, 1; TB, 1	
Yamamoto et al.,												
Zhang et al.,											Syphilis, 1	
Zhao et al.,											HCV, 1	
Zhu et al.,										DM, 1		Smoke, 1

Note: CKD=Chronic kidney disease; COPD=Chronic obstructive pulmonary disease; CVD=Cardiovascular disease; DM; Diabetes mellitus; ESRD=End stage renal disease; HBV= Hepatitis B virus infection; HCV=Hepatitis C virus infection; HLD; Hyperlipidaemia; HPT=Hypertension; NS=Non-specific; SOT=Solid organ transplant; TB=Tuberculosis;

Table S6: **HIV profile of patients with HIV+Covid-19**														
**Author**	**Year of HIV diagnosis**	**Duration of HIV infection by 2020, year**	**Year started on ART**	**Duration of on antiretroviral treatment, year**	**Received ART treatment, n**	**On ART treatment**	**Nadir CD4+ count, cell/ul**	**Summary of CD4 counts before admission**	Advanced HIV/AIDS diagnosis according to WHO	**CD4+ T-cell counts, cell/ul (Before admission, mean)**	**CD4% (Before admission)**	**CD4:CD8 ratio (Before admission, mean)**	**Viral load (Before admission, mean)**	**ART regimen before admission**
Adachi et al.			Unknown, 2 (100)	Unknown, 2 (100)	Yes, 2 (100)			≥350, 2 (100)	*No, 2 (100)*	668.0			Unknown, 2 (100)	INSTI, 2 (100); NRTI, 2 (100)
Altuntas et al.	2008 - 2010	11.0	Unknown, 4 (100)	2; Unknown, 4 (80)	Yes, 4 (100)	No, 1 (25)		≥350, 1 (25), Unknown, 3 (75)	No, 1 (25); Unknown, 3 (75)	1385.0			<20, 2 (50); Unknown, 2 (50)	INSTI, 3 (75); NRTI, 3 (75); None, 1 (25)
Baluku et al.			2015	5.0	Yes, 1 (100)			Unknown, 1 (100)	Unknown, 1 (100)				Unknown, 1 (100)	Tenofovir disoproxil fumarate, lamivudine, Efavirenz
Benkovic et al,	1988 - 2006	23.8	Unknown, 4 (100)	Unknown, 4 (100)	Yes, 4 (100)			>350, 4 (100)	Yes, 1 (25); No, 3 (75)	1085.3			<20, 3 (75); Non-UD, 1 (25)	INSTI, 3 (75); NNRTI, 1 (25); NRTI, 4 (100); CCR5 receptor, 1 (25)
Blanco et al.	2003 - 2020	10.8	Unknown, 5 (100)	Unknown, 5 (100)	Yes, 5 (100)			<350, 1 (20); ≥350, 4 (80)	Yes, 1(20); No, 4 (80)	563.6		0.7	Non-UD, 5 (100)	PI, 2 (40); INSTI, 2 (40); NRTI, 4 (80); None, 1 (20)
Boulle et al.			Unknown, 3978	Unknown, 3978				Unknown, 3978 (100)	Yes, 188 (4.7);	<200, 70			Unknown, 3978	Unknown, 3978 (100)

			(100)	(100)					Unknown, 3790 (95.3)				(100)	
Byrd et al.	1980 - 2017	12.4	Unknown, 27 (100)	Unknown, 27 (100)	Yes, 27 (100)			<350, 6 (22.2); ≥350, 21 (80.8)	Yes, 6 (22.2); No, 21 (80.8)	638.4	*34.3*		<20, 25 (92.6); Non-UD, 2 (7.4)	INSTI, 24 (88.9); NNRTI, 3 (11.1); NRTI, 26 (96.3)
Calza et al. b		14.6 (IQR=8.1-23.4)	Unknown, 14 (100)	Unknown, 14 (100)	Yes, 14 (100)		259	612 (Range=339-886)	Unknown, 14 (100)	612 (IQR=339-886)		0.69 (IQR=0.48-0.91)	Non-UD, 14 (100)	NNRTI, 5; Booster PI, 4 ; INSTIs, 5
Calza et al. a		16.2 (IQR=8.4-25.3)	Unknown, 26 (100)	Unknown, 26 (100)				566 (Range=304-821)	Unknown, 26 (100)	566 (IQR=304-821)			<50, 22 (84.6); Unknown, 4 (15.4)	NNRTIs, 13; Booster PI, 6; INSTIs, 11; tenofovir disoproxil fumarate or tenofovir alafenamide, 16
Chen et al,	2018	2.0	2018	2.0	Yes, 1 (100)			Unknown, 1 (100)	Unknown, 1 (100)				Unknown, 1(100)	Tenofovir 0.3g, lamivudie 0.3g, efavirenz 0.6g
Chiappe et al.			Unknown, 1 (100)	Unknown, 1 (100)				≥350, 1 (100)	No, 1 (100)	438.0			<20, 1 (100)	Emtricitabine/tenofovir disoproxil fumarate, atazanavir/ritonavir
Childs et al.		14.6	Unknown, 18 (100)	Unknown, 18 (100)			97	395(Range=238-680)	Unknown, 18 (100)	395(IQR=238-680)			<50, 17 (94.4); Unknown, 1(5.6)	PI, 11; INSTI, 3; NNRTI, 4; NRTI, 18
Cipolat and Sprinz	2005	15.0	Unknown, 1 (100)	Unknown, 1 (100)				≥350, 1 (100)	No, 1 (100)	426.0		1.3	<20, 1 (100)	Tenofovir disoproxil fumarate, lamivudine, dolutegravir
Coleman et al.	2006	14.0	Unknown, 1 (100)	Unknown, 1 (100)	Yes, 1 (100)			≥350, 1 (100)	No, 1 (100)	422.0	35.6		<20, 1 (100)	Emtricitabine/tenofovir disoproxil 200/245 mg, raltegravir 1200 mg once daily
Collins et al.			Unknown, 20 (100)	Unknown, 20 (100)	Yes, 20 (100)	Yes, 19 (95); Not, 1 (5)		426 (Range=262-815)	No	425 (IQR=262-815)	29 (range=21-36)		<200, 18 (90); 200-1000, 1 (5); >1000, 1 (5)	Bictegravir/emtricitabine/tenofovir aladenamide, 7; dolutegravir/abacavir/lamivudine, 5; NNRTS, 2; PI, 4; INSTI, 16
Dandachi et al.		<1, 14; 1-5 years, 37; >5, 180	Unknown, 286 (100)	Unknown, 286 (100)	Yes, 263 (92			≥350, 286(100)	No, 286 (100)	531.0			<200, 235 (82.2); Unknown, 51 (17.8)	INI+2 NRTI, 171; NNRTI+2NRTI, 20; PI+2 NRTI, 20; dual ART regimen, 222; not on ART, 16
Del Amo et al.			Unknown, 236 (100)	Unknown, 236 (100)				Unknown, 236 (100)	Unknown, 236 (100)				Unknown, 236(100)	Tenofovir disoproxil/emtricitabine, 21; tenofovir

														alafenamide/emtricitabine, 100; abacavir/lamivudine, 57; other regimen, 58; Third drug is NNRTI, 36; third drug is PI, 34; third drug is INSTI, 143
Di Biagio et al. a	1999 - 2015	13.3	2002-2015	12.5				≥350, 4 (100)	No, 4 (100)	766.3		0.7	<3, 4 (100)	PI, 1 (25); INSTI, 1 (25); NNRTI, 3 (75); NRTI, 3 (75)
Di Biagio et al, b		13.5	Unknown, 69 (100)	9.0			283	Unknown, 69 (100)	Yes, 32 (46.4); Unknown, 37 (53.6)			0.85 (IQR=nil-nil)	<50, 61 (88.4); Unknown, 8 (11.6)	PI, 17; NNRTI, 23; INSTI, 32; Tenofovir, 41
Di Giambenedetto et al,	1997	23.0	Unknown, 1 (100)	Unknown, 1 (100)			159	≥350, 1 (100)	No, 1 (100)	709.0			<20, 1 (100)	Rilpivirine/emtricitabine/tenofovir alafenamide
D'Ettorre et al.	1997	23.0	Unknown, 1 (100)	Unknown, 1 (100)				<350, 1 (100)	Yes, 1 (100)	242.0		0.7	<37, 1 (100)	Darunavir/cobicistat
Elhadi et al.			Unknown, 1 (100)	Unknown, 1 (100)	Yes, 1 (100)	Not, 1 (100)		Unknown, 1 (100)	Yes, 1 (100)				Unkown, 1 (100)	Zidovudine 300 mg
Etienne et al,			Unknown, 54 (100)	Unknown, 54 (100)	Yes, 54 (100)		215 (IQR=100-340)	584 (Range=474-773)	Unknown, 54 (100)	583 (IQR=474-773)	33.1 (range=26-40.8)		<40, 51 (94.4); Unknown, 3 (5.6)	Darunavir, 6 ; atazanavir, 2; Lopinavir, 1; NNRTI, 25; NRTI,43; INSTI, 33
Faranacci et al,	1988	30.0	Discontinued for 10 years	20.0	Yes but disconti-nued for 10 years	Not, 1 (100)		<350, 1 (100)	Yes, 1 (100)	10.0		0.0	Unknown, 1 (100)	None, 1 (100)
Gadelha et al.			Unknown, 2 (100)	Unknown, 2 (100)	Yes, 2 (100)	Not, 2 (100)		<350, 1 (50); ≥350, 1 (50)	Yes, 2 (100)	276.0			151,184	Unknown, 2 (100)
Geretti et al.			Unknown, 115 (100)	Unknown, 115 (100)	Yes, 103 (89.6)			Unknown, 115 (100)	Yes, 9 (7.8); Unknown, 106 (92.2)				Unknown, 115 (100)	Unknown, 115 (100)
Gervasoni et al.			Unknown, 47 (100)	Unknown, 47 (100)				≥ 350, 47 (100)	No, 47 (100)	636.0			<20, 44 (93.6); Non-UD, 3 (6.4)	Tenofovir alafenamide/emtricitabine/ bictegravir, 10; abacavir/ lamivudine, INSTI, 6; dolutegravir/lamivudine, 5;

														dolutegravir, boosted PI, 5; tenofovir lafenamide/emtricitabine, boosted PI, 5; other regimen, 6
Gudipati et al.			Unknown, 14 (100)	Unknown, 14 (100)				<350, 4 (28.6); ≥350, 10 (71.4)	Yes, 4 (28.6); No, 10 (71.4)	612.1			<20, 12 (100)	Unknown, 14 (100)
Guo et al.			Unknown, 14 (100)	Unknown, 14 (100)	No, 1 (7.1)			<350, 6 (42.9) ≥350, 8 (57.1)	Yes, 6 (42.9); Unknown, 8 (57.1)	408.5			<20, 11 (78.6); Non-UD, 2 (14.3); Unknown, 1 (7.1)	PI, 1 (7.1); INSTI, 1 (7.1); NNRTI, 11 (78.6); NRTI, 13 (92.9)
Haddad et al.			Unknown, 1 (100)	Unknown, 1 (100)	Yes, 1 (100)	Yes, 1 (100)		≥350, 1 (100)	Yes, 1 (100)	604.0			<20, 1 (100)	Dolutegravir/lamivudine
Hadi et al.			Unknown, 404 (100)	Unknown, 404 (100)	Yes, 284 (70.3)			Unknown, 404 (100)	Unknown, 404 (100)				Unknown, 404 (100)	Unknown, 404 (100)
Harter et al.	1989-2019	11.5	1992-2020	9.3	Yes, 33 (100)			<350, 4 (12.1); ≥350, 28 (84.8); Unknown, 1 (3.1)	Yes, 4 (12.1); No, 29 (87.9)	695.3		1.0	<50, 30 (90.9); >50, 2 (6.1); Unknown, 1 (3.0)	PI, 4 (12.1); INSTI, 20 (60.6); NNRTI, 10 (30.3); NRTI, 31 (96.9)
Ho et al.		20.0	Unknown, 93 (100)	Unknown, 93 (100)	Yes, 89 (95.7)		320	555 (Range=339–752)	Unknown, 93 (100)	554 (IQR=339–752)	33		<50, 57 (61.3); Unknown, 36 (38.7)	Tenofovir, 62; PI (lipinarivr, atazanavir, darunavir), 12;
Hu et al.		6.0	Unknown, 12 (100)	4.6	Yes, 10 (83.3)			≥350, 12 (100)	Yes, 1 (8.3); No, 11 (91.7)	555.0			<20, 8 (66.7); Unknown, 4 (33.3)	Tenofovir disoproxil fumarate+efavirenz+lamivudine, 5; zidovudine+nevirapine+lamivudine, 3; zidovudine+efavirenz+lamivudine, 2; Tenofovir disoproxil fumarate+lopinavir/ritonavir+lamivudine, 2
Huang et al.		3 and below,	Unknown, 35 (35)	Unknown, 35 (35)	Yes, 32 (91.4)	Yes, 28		<350, 5 (14.3); ≥350,	Yes, 5 (14.3);	<50, 1; 50-199, 4; 200-			<20, 22 (62.9);	NRTI, 32; NNRTI, 30; PI, 1; INSTI, 1

		13; >3, 22				(80)		15 (42.9); Unknown, 15 (42.9)	Unknown, 30 (85.7)	499, 14; 500 onward 15			Unknown, 13 (37.1)	
Inciarte et al.		11 (7-16)	Unknown, 53 (100)	4.6	Yes, 51 (96.2)		303 (140-434)	≥350, 624 (100)	Yes, 1 (1.9); No, 52 (98.1)	624 (IQR=462-838)		0.9 (IQR=0.66-1.2)	<50, 49 (92.5); Unknown, 4 (7.5)	Any, 51; Triple therapy, 44; INSTI-based, 29; Pi-based, 15; NNRTI-based, 13; tenofovir (TAF or TDF), 35, abacavir, 9
Iordanou et al.	1995	25.0	Unknown, 1 (100)	Unknown, 1 (100)				≥350, 1 (100)	No, 1 (100)	1640.0			<20, 1 (100)	Elvitegravir, cobicistat, Emtricitabine, Tenofovir alafenamide fumarate
Isernia et al.			Unknown, 24 (100)	Unknown, 24 (100)				<350, 4 (16.7); ≥350, 20 (83.3)	Yes, 4 (16.7); Unknown, 20 (83.3)	627.6			<20, 20 (83.3); Non-UD, 4 (16.7)	PI, 5 (20.8); INSTI, 19 (79.2); NNRTI, 7 (29.2); NRTI, 22 (91.7)
Karmen et al.			Unknown, 21 (100)	15.0	Yes, 21 (100)			299 (Range=135-542)	Unknown ,21 (100)	298 (135-542)	24 (range=16-28)		<50, 17 (81); Unknown, 4 (19)	Unknown, 21 (100)
Khaba et al.	2020	0.0	Naïve	Naïve	Naïve, 1 (100)			<350, 1 (100)	Yes, 1 (100)	17.0			1487946	Naïve, 1 (100)
Kim et al.			2003	7.0	Yes, 1 (100)			350, 1 (100)	No, 1 (100)	555.0		0.4	<20, 1 (100)	Elvitegravir/cobicisat/emtricitabine/Tenofovir
Kumar et al.	1997	23.0	1997	23.0				350, 1 (100)	No, 1 (100)	395.0	28	0.7	<20, 1 (100)	Dolutegravir, emtricitabine, tenofovir alafenamide.
Li et al.			Naïve, 2 (100)	Naïve, 2 (100)	Naïve, 2 (100)			Unknown, 2 (100)	Unknown, 2 (100)				Unknown, 2 (100)	Naïve, 2 (100)
Liu et al,					Yes, 12 (60)			<350, 20 (100)	Yes, 20 (100)	237 (IQR=142.5-346.8)		0.4 (IQR=0.2-0.6)	Unknown, 20 (100)	NRTI, 12; NNRT, 6; PI, 8
Madge et al.		8 years to 31 years					<200, 10	440 (Range=239-651)	Unknown, 18 (100)	439 (IQR=239-651)			<40, 18 (100)	Truvada or descovy, 7; abacavir/lamivudine with NNRT, 4; INSTI, 11; PI, 5
Maggiolo et al,							281	≥350, 55 (100)	No, 55 (100)	904.0		0.9	<50, 54 (98.2); Unknown, 1 (1.8)	NRTI 47; NNRTI, 20; PI, 11; INSTI, 32
Mang et al.		Naïve	Naïve, 1 (100)	Naïve, 1 (100)	Naïve, 1 (100)			Unknown, 1 (100)	Unknown, 1 (100)				Unknown, 1 (100)	Naïve, 1 (100)
Marimuthu et al.	2005 - 2017	10.4	Unknown, 6 (100)	7.8; Naïve, 1 16.7)	Yes, 5 (83.3); Naïve,			<350, 1 (16.7); ≥350, 5 (83.3)	Yes, 2 (33.3); No, 4	545.2			Unknown, 6 (100)	PI, 1 (16.7); NNRTI, 4 (66.7); NRTI, 5 (71.4); Naïve, 1 (16.7)

					1 (16.7)				(66.7)					
Meyerowitz et al.			Unknown, 36 (100)	Unknown, 36 (100)	No, 1 (2.8)			<350, 8 (22.2); ≥350, 28 (77.8)	Yes, 8 (22.2); No, 28 (77.8)	691.7	30.691667		Unknown, 36 (100)	PI, 4 (11.1); INSTI, 28 (77.8); NNRTI, 10 (27.8); NRTI, 34 (94.4); None, 1 (2.8)
Miyashita and Kuno			Unknown, 161 (100)	Unknown, 161 (100)				Unknown, 161 (100)	Unknown, 161 (100)				Unknown, 161 (100)	Unknown, 161 (100)
Modi et al,	2010	10.0	2010	10.0	Yes, 1 (100)			Unknown, 1 (100)	Unknown, 1 (100)				Unknown, 1 (100)	Efavirenz, emtricitabine, tenofovir disoproxil fumarate. Changed to raltegravir, emtricitabine, tenofovir disoproxil fumarate after liver transplant.
Molina et al.			Unknown, 8 (100)	Unknown, 8 (100)				<350, 1 (12.5); ≥350, 7 (87.5)	Yes, 1 (12.5); No, 7 (87.5)	568.4			<20, 6 (75); Non-UD, 2 (25)	PI, 3 (37.5); INSTI, 4 (50); NNRTI, 1 (12.5); NRTI, 8 (100)
Mondi et al.	1998 - 2019	11.5	Unknown, 5 (100)	8.3	Yes, 4 (80)			Unknown, 5 (100)	Yes, 5 (100)		43	0.7	< 30, 5 (100)	PI, 2 (40); INSTII, 3 (60); NNRTI, 1 (20); NRTI, 3 (60)
Nakamoto et al.	2018	2.0	Naïve, 1, (100)	Naïve, 1, (100)	Naïve, 1 (100)			≥350, 1 (100)	No, 1 (100)	491.0			12800	Naïve, 1 (100)
Okoh et al.			Unknown, 27 (100)	Unknown, 27 (100)				552 (Range=286-710)	Unknown, 27 (100)	551 (IQR=286-710)	29 (range =20-35)	0.9 (IQR=0.5 -1.0)	<20, 11; 20-120, 15; >120, 1	INSTI, 9; INSTI/PI, 5 ; NNRTI, 5 ; NNRTI/INSTI, 3 ; PI, 1 ; N/A, 4
Parker et al. a			2016	4.0	Yes, 1 (100)			Unknown, 1 (100)	Unknown, 1 (100)				<20, 1 (100)	Emtricitabine, tenofovir, efavirenz
Parker et al. b			Unknown, 24 (100)	Unknown, 24 (100)	Yes, 22 (91.7); No, 2 (8.3)	Yes, 18 (75); No, 6 (25)		<350, 24 (100)	Yes, 24 (100)	325.0			Unknown, 1 (100)	Unknown, 24 (100)
Patel and Pella			Unknown, 1 (100)	Unknown, 1 (100)				≥350, 1 (100)	No, 1 (100)	497.0	43		Unknown, 1 (100)	Emtricitabine, tenofovir, atazanavir, ritonavir
Przydzial et al.			Unknown, 2 (100)	Unknown, 2 (100)	Not, 2 (100)			<350, 2 (100)	Yes, 2 (100)	63.3			1283450	None, 2 (100)
Qasim et al.			Unknown, 1 (100)	Unknown, 1 (100)	Yes, 1 (100)	No, 1 (100)		Unknown, 1 (100)	Unknown, 1 (100)				517	Bictegravir/emtricitabine/tenofovir alafenamide, atovaquone,

														prochlorperzine, ondansetron, tramadol
Ridgway et al.			Unknown, 5 (100)	Unknown, 5 (100)	Yes, 5 (100)	Yes, 4 (80); No, 1 (20)		<350, 4 (80); ≥350, 1 (20)	Yes, 4 (80); No, 1 (20)	330.6			<20, 4 (80); Non-UD, 1 (20)	PI, 2 (40); INSTI, 5 (100); NRTI, 5 (100)
Riva et al.			Unknown, 3 (100)	Unknown, 3 (100)	Yes, 1 (33.3)			≥350, 2 (66.7); Unknown, 1 (33.3)	No, 2 (66.7); Unknown, 1 (33.3)	592.0			<20, 2 (66.7); Unknown, 1 (33.3)	PI, 3 (100); NRTI, 2 (66.7)
Rivas et al.			Unknown, 2 (100)	Unknown, 2 (100)	No, 1 (50)			Unknown, 2 (200)	Yes, 2 (100)				Unknown, 2 (100)	None, 1 (100); Unknown, 1 (100)
Ruan et al,	2015 - 2019	2.8	Unknown, 4 (100)	Unknown, 4 (100)	Yes, 2 (50)			<350, 2 (50); Unknown, 2 (50)	Yes, 2 (50); Unknown, 2 (50)	< 50, 2			Unknown, 4 (100)	Unknown, 4 (100)
Sasset et al.	1990	30.0	Unknown, 2 (100)	Unknown, 2 (100)	Yes, 2 (100)	Yes, 2 (100)		<350, 1 (50); ≥350, 1 (50)	Yes, 1 (50); No, 1 (50)	319.0	28.7		< 40, 2 (100)	Unknown, 2 (100)
Shalev et al.			Unknown, 31 (100)	Unknown, 31 (100)	Yes, 31 (100)			≥350, 31 (100)	No, 31 (100)	396.0	28.7		<37, 28 (90.3); <200, 3 (9.7)	INSTI+2 NRTI, 20; NNRTI+2 NRTI, 3; PI/b+2NRTI, 4 ; Other, 4 ; Contain TDF/TAF, 17 ; Contain PI, 7
Shekhar et al.			Unknown, 5 (100)	Unknown, 5 (100)				<350, 1 (20); ≥350, 3 (60); Unknown, 1 (20)	Yes, 1 (20); No, 3 (60); Unknown, 1 (20)	602.5			<20, 2 (40); Unknown, 3 (60)	INSTI, 5 (100); NRTI, 5 (100)
Sigel et al.			Unknown, 88 (100)	Unknown, 88 (100)	Yes, 88 (100)			<350, 7 (8); ≥350, 20 (22.7); Unknown, 61 (69.3)	Yes, 7 (8); No, 20 (22.7); Unknown, 61 (69.3)	<50, 1; 50-200, 6; 201-500, 19; >500, 20			<50, 66 (75); >50, 16 (18.2); Unknown, 6 (6.8)	INSTI, 69; PI, 15; NNRTI, 8; NRTI, 85
Stoeckle et al.			Unknown, 30 (100)	Unknown, 30 (100)	Yes, 29 (100)			333 (Range=123–526)	Unknown, 30 (100)	332 (IQR=123-526)		0.7 (IQR=0.3 -1.0)	<20, 30 (100)	PI, 6; Non PI, 23* detailed in paper
Su et al.	2008	12.0	Unknown, 1 (100)	Unknown, 1 (100)	Yes, 1 (100)		294	≥350, 1 (100)	No, 1 (100)	430.0			Unknown, 1 (100)	Zidovudine (300mg twice/day), lamivudine (300 mg once/day), efavirenz (600 mg once/night)

Sun et al.	2010	10.0	Unknown, 1 (100)	Unknown, 1 (100)	Yes, 1 (100)	Yes, 1 (100)	201	≥350, 1 (100)	No, 1 (100)	900.0	36		<20, 1 (100)	Tenofovir, lamivudine and efavirenz on 2010. Efavirenz then was switched to rilpivirin in 2017.
Suwanwongse and Shabarek, a			Unknown, 9 (100)	Unknown, 9 (100)	Yes, 8 (88.9); No, 1 (11.1)	Yes, 6 (66.7) o, 2 (22.2)		<350, 2 (22.2); ≥350, 6 (66.7); Unknown, 1 (11.1)	Yes, 2 (22.2); No, 6 (66.7); Unknown, 1 (11.1)	616.5			<20, 5 (55.6); Non-UD, 3 (33.3); Unknown, 1 (11.1)	PI, 2 (22.2); INSTI, 6 (66.7); NNRTI, 1 (11.1); NRTI, 8 (88.9); None, 1 (11.1)
Suwanwongse and Shabarek, b			Unknown, 5 (100)	Unknown, 5 (100)	Yes, 4 (80); No, 1 (20)	Yes, 4 (80)		<350, 2 (40); ≥350, 2 (40); Unknown, 1 (20)	Yes, 2 (40); No, 2(40); Unknown, 1 (20)	587.0			<20, 2 (40); Non-UD, 2 (40); Unknown, 1 (20)	PI, 1 (20); INSTI, 3 (60); NNRTI, 2 (40); NRTI, 4 (80), None, 1 (20)
Toombs et al.	2002 - 2013	11.0	Naïve, 1 (33.3); Unknown, 2 (66.7)	Naïve, 1 (33.3); Unknown, 2 (66.7)	Yes, 2 (66.7); Naïve, 1 (33.3)			<350, 2 (66.7); ≥350, 1 (33.3)	Yes, 2 (66.7); No, 1 (33.3)	373.3		0.4	<20, 2 (66.7); Non-UD, 1 (33.3)	INSTI, 1 (33.3); NNRTI, 1 (33.3); NRTI, 2 (66.7); Naïve, 1 (33.3)
Vizcarra et al,		16.5	Unknown, 35 (100)	Unknown, 35 (100)			174	≥350, 35 (100)	No, 35, (100)	502.0		0.8	<50, 34 (97.1); Unknown, 1 (2.9)	PI, 7; NNRTI, 3 ; INSTI, 30; Tenofovir,24
Wang et al.			Unknown, 2 (100)	Unknown, 2 (100)				<350, 1 (100)	Yes, 1 (100)	34.0		0.1	Unknown, 1 (100)	Unknown, 1 (100)
Wu et al. a	2012	8.0	Unknown, 2 (100)	Unknown, 2 (100)	Yes, 1 (100)	Yes, 1 (100)	224	≥350, 1 (100)	No, 1 (100)	Normal			<20, 1 (100)	Efavirenz 600 mg, zidovudine 300 mg, lamivudine 150mg
Wu et al., b	2014 - 2020	3.0	2018, 1 (50); Naïve, 1 (500	2; Naïve, 1 (50)	Yes, 1 (100); Naïve; 1 (100)			Unknown, 2 (200)	Yes, 1 (50), Unknown, 1 (50)				Unknown, 2 (100)	NNRTI, 1 (50); NRTI, 1 (50); Naïve, 1 (50)
Yamamoto et al.			Unknown, 5 (100)	Unknown, 5 (100)	Yes, 5 (100)			<350, 2 (4); ≥350, 3 (60)	Yes, 2 (40); No, 3 (60)	454.4			<20, 5 (100)	INSTI, 5 (100); NRTI, 5 (100)
Zhang et al.	2020	0.0	Naïve, 2 (100)	Naïve, 2 (100)	Naïve, 2 (100)			Unknown, 2 (100)	Unknown, 2 (100)				Unknown, 2 (100)	Naïve, 2 (100)
Zhao et al,	2016	4.0	Unknown, 1 (100)	Unknown, 1 (100)	Yes, 1 (100)			<350, 1 (100)	Yes, 1 (1000	25-300			<20, 1 (100)	Lamivudine, tenofovir, efavirenz

Zhu et al.			Unknown, 1 (100)	Unknown, 1 (100)				Unknown, 1 (100)	Unknown, 1 (100)		4.75		Unknown, 1 (100)	Unknown, 1 (100)

Note: Data are presented either in n, (%), or mean (range) or median (IQR). INSTI=Integrase strand transfer inhibitor; NNRTI=Non-nucleoside reverse transcriptase inhibitor; NRTI=Nucleoside reverse transcriptase inhibitor; PI=Protease inhibitor; UD=Undetectable viral load which it is defined as <20 copies/ml;

Table S7: **Laboratory results, chest imaging findings and vital signs among HIV+Covid-19**

**Author**	**Diagnosis methods**	**Summary of positive result by different diagnosis methods**	**Chest imaging findings**			**Vital signs**						
			**Pattern of lesion**	**Predominant distribution of lesion**	**Laterality of lesion**	**Temperature(^{o}C)**	**Systolic (mmHg)**	**Diastolic (mmHg)**	**Breath per minutes**	**Heart rate, beats per min**	**O2 saturation in ambient air, %**	**PaO2/FiO2 ration mmHg**
Adachi et al.	Nucleic acid-based testing, 2 (100)	Nucleic acid-based testing, 2 (100)	GGO, 2 (100);	Patchy, 1 (50); Widespread, 1 (50)	Unilateral, 1 (50); Bilateral, 1 (50)							
Altuntas et al,	Nucleic acid-based testing, 4 (100)	Nucleic acid-based testing, 4 (100)	GGO, 4 (100)	Basal, 1 (25); Patchy, 1 (25); Widespread, 2 (50); Peripheral, 1 (25)	Bilateral, 4 (100)	38					93.5	
Baluku et al.	Nucleic acid-based testing, 1 (100)	Nucleic acid-based testing, 1 (100)	Normal, 1 (100)			36.4	110	80	26	84	96	
Benkovic et al.	Nucleic acid-based testing, 4 (100)	Nucleic acid-based testing, 4 (100)	Normal, 4 (100)			38.3	124.5	68		79	95	
Blanco et al,	Nucleic acid-based testing, 5 (100)	Nucleic acid-based testing, 5 (100)	Normal, 2 (40); GGO, 1 (20); Infiltrate, 1 (20); Pleural effusion, 1 (20)	Basal, 2 (40); Interstitial, 1 (20)	Unilateral, 1 (20); Bilateral, 2 (40)	39	124.2	72.2	20.4	97.2	97	206
Boulle et al.	Nucleic acid-based testing, 3978 (100)	Nucleic acid-based testing, 3978 (100)										
Byrd et al.	Nucleic acid-based testing, 27 (100)	Nucleic acid-based testing, 27 (100)	Air space, 12 (44.4); GGO, 1 (3.7)	Basal, 1 (3.7)	Unilateral, 2 (7.4); Bilateral, 7(25.9)						91.9	
Calza et al. b	Nucleic acid-based testing, 14 (100)	Nucleic acid-based testing, 14 (100)										<300, 1
Calza et al. a	Nucleic acid-based testing, 26 (100)	Nucleic acid-based testing, 26 (100)							>20, n=4		<95, 7;	Initial respira-tory failure <300, 2
Chen et al.	Nucleic acid-based testing, 1 (100)	Nucleic acid-based testing, 1 (100)	Interlobar fissure	Patchy, 1 (100)	Interlobar, 1 (100);							

			thickening, 1 (100); Consolidation, 1 (100)		Unilateral, 1 (100)							
Chiappe et al.	Nucleic acid-based testing, 1 (100)	Nucleic acid-based testing, 1 (100)	GGO, 1 (100); Infiltrate, 1 (100)	Patchy, 1 (100); Alveolar, 1 (100)	Bilateral, 1 (100)							
Childs et al.	Nucleic acid-based testing, 18 (100)	Nucleic acid-based testing, 18 (100)	Infiltrates, 13 (72.2)									
Cipolat and Sprinz	Nucleic acid-based testing, 1 (100)	Nucleic acid-based testing, 1 (100)	Opacities, 1 (100)		Interlobar, 1 (100); Bilateral, 1 (100)	39					98.2	
Coleman et al.	Nucleic acid-based testing, 1 (100)	Nucleic acid-based testing, 1 (100)	GGO, 1 (100); Infiltrate, 1(100)	Apical, 1 (100); Subpleural, 1 (100); Interstitial, 1 (100)	Bilateral, 1(100)							
Collins et al.	Nucleic acid-based testing, 20 (100)	Nucleic acid-based testing, 20 (100)	GGO, 5 (25)); Infiltrates, 2 (10)	Diffuse, 5 (25); Focal, 1 (5); Basal, 5 (25)	Bilateral, 5 (25)	37.9 (37.2-38.7)			20 (18-21)		96 (84-97)	
Dandachi et al.	Nucleic acid-based testing, 285 (99.7); Serological testing, 1 (0.3)	Nucleic acid-based testing, 285 (100); Serological testing, 1 (100)	GGO, 29 (10.1); Consolidation, 6 (2.1)	Patchy, 29 (10.1); Interstitial, 6 (2.1) Multifocal, 29 (10.1)	Unilobar, 6 (2.1)						<94, 72;	
Del Amo et al.	Nucleic acid-based testing, 236 (100)	Nucleic acid-based testing, 236 (100)										
Di Biagio et al, a	Unknown, 4 (100)	Unknown diagnosis method and unknown result, 4 (100)	GGO, 4 (100); Infiltrates, 4 (100)	Patchy, 4 (100); Alveolar, 4 (100)	Bilateral, 4 (100)							
Di Biagio et al. b	Nucleic acid-based testing, 69 (100)	Nucleic acid-based testing, 69 (100)										
Di Giambenedetto et al.	Nucleic acid-based testing, 1 (100)	Nucleic acid-based testing, 1 (100)	GGO, 1 (100); Consolidation, 1 (100)		Bilateral, 1 (100)							
D'Ettorre et al.	Nucleic acid-based testing, 1 (100)	Nucleic acid-based testing, 1 (100)	GGO, 1 (100); Crazy paving pattern, 1 (100)	Peripheral, 1 (1)	Bilateral, 1(100)	37.5	120	70	24	120	96	333
Elhadi et al.	Nucleic acid-based testing, 1 (100)	Nucleic acid-based testing, 1 (100)	Infiltrate, 1 (100); GGO, 1 (100)	Basal, 1 (100); Peripheral/ subpleural, 1 (100)	Bilateral, 1(100)	38.5	130	80	24	110	77	

Etienne et al	Unknown, 54 (100)	Diagnosis method unknown but reported as positive, 38 (70.4)										
Faranacci et al.	Nucleic acid-based testing, 1 (100)	Nucleic acid-based testing, 1 (100)	GGO, 1(100)									50
Gadelha et al.	Nucleic acid-based testing, 2 (100)	Nucleic acid-based testing, 2 (100)	GGO, 2 (100)		Bilateral, 2 (100)	38			18	93.5	94.5	
Geretti et al.	Nucleic acid-based testing or serological or imaging finding or symptoms or contact history, 115 (100)	Unknown result, 115 (100)	Infiltrates, 46 (40)			37.8	130	80		97		
Gervasoni et al.	Either nucleic acid-based testing, epidemiological risk factor or Imaging finding	Nucleic acid-based testing, 28 (59.6); Serological testing, 2 (4.3); Epidemiological risk factor, 21 (44.7); Imaging finding, 4 (8.5)										
Gudipati et al.	Nucleic acid-based testing, 14 (100)	Nucleic acid-based testing, 14 (100)										
Guo et al.	Nucleic acid-based testing, 14 (100)	Nucleic acid-based testing, 12 (85.7);	GGO, 9 (75); Shadowing, 2 (16.7); Pleural effusion, 1 (8.3)	Patchy, 2 (16.7)	Bilateral, 3 (25)	38.7	126.4	81.4			92.4	
Haddad et al.	Unknown, 1 (100)	Unknown diagnosis method but reported as positive, 1 (100)	GGO, 1 (100)	Diffuse, 1 (100); Patchy, 1 (100); Basal, 1 (100); M Nodular, 1 (100)	Unilateral , 1 (100)	38						
Hadi et al.	Unknown, 404 (100)	Unknown diagnosis method and unknown test result, 404 (100)										
Harter et al.	Nucleic acid-based testing, 33 (100)	Nucleic acid-based testing, 33 (100)										
Ho et al.	Nucleic acid-based testing, 93 (100)	Nucleic acid-based testing, 93 (100)									<92, 43	
Hu et al.	Unknown, 12 (100)	Unknown diagnosis method and unknown test results, 12 (100)										
Huang et al.	Nucleic acid-based testing, 35 (100)	Nucleic acid-based testing, 35 (100)										

Inciarte et al.	Nucleic acid-based testing, 53 (100)	Nucleic acid-based testing, 42 (79.2)	GGO, 8 (19); Consolidation, 1 (2.4)	Interstitial, 12 (28.6); Alveolar, 7 (16.7)	Bilateral, 18 (42.9)		121 (119-132)	74 (70-80)	18 (16-23)	88 (80-96)	98 (96-99)	
Iordanou et al.	Nucleic acid-based testing, 1 (100)	Nucleic acid-based testing, 1 (100)	Consolidation, 1 (100)		Bilateral, 1 (100)	38	117	72	22	105	92	328
Isernia et al.	Nucleic acid-based testing, 24 (100)	Nucleic acid-based testing, 24 (100)										
Karmen et al.	Nucleic acid-based testing, 21 (100)	Nucleic acid-based testing, 21 (100)			Bilateral, 18 (94.7); unilateral, 1 (5.3)	37.6 ± 0.8	126 (115-139)	74.4 ± 11	20 (18-25)			
Khaba et al.	Nucleic acid-based testing, 1 (100)	Nucleic acid-based testing, 1 (100)	Infiltrates, 1 (100)		Bilateral, 1 (100)	38.7	95	33		103	95	
Kim et al.	Nucleic acid-based testing, 1 (100)	Nucleic acid-based testing, 1 (100)	Consolidation, 1 (100); air bronchogram, 1 (100); GGO, 1 (100); Halo sign, 1 (100)	Multifocal, 1 (100); Basal, 1 (100)	Bilateral, 1 (100)	36.8	117	77	16	72	100	
Kumar et al.	Nucleic acid-based testing, 1 (100)	Nucleic acid-based testing, 1 (100)				38.3	172	95		108	100	
Li et al.	Nucleic acid-based testing, 2 (100) Serological testing 2 (100)	Nucleic acid-based testing, 1 (50); Serological, 1 (50)	Consolidation, 2 (100)			37.7	135	90.5	31	110.5		
Liu et al.	Nucleic acid-based testing, 20 (100); Serological testing, 20 (100)	Nucleic acid-based testing, 19 (95); Serological testing, 12 (60)	GGO, 11 (35.5); Consolidation, 6 (19.4); Mixed pattern, 1 (3.2), Fibrotic streaks, 5 (16.1); Subpleural transparent line, 1 (3.2); Air bronchogram, 3 (9.7); Bronchial distortion, 2 (6.5); Pleural retraction, 3 (9.7)									
Madge et al.	Nucleic acid-based testing, 16 (88.9);	Nucleic acid-based testing, 16 (100);										

	Imaging finding and contact history, 2 (11.1)	Imaging finding and contact history, 2 (100)										
Maggiolo et al.	Nucleic acid-based testing, 16 (29.1); Serological test, 33 (60); Symptoms, 6 (10.9)	Nucleic acid-based testing, 16 (100); Serological test, 33 (100); Symptoms, 6 (100)										
Mang et al.	Unknown, 1 (100)	Unknown diagnosis method and unknown results, 1 (100)	GGO, 1 (100); Consolidation, 1 (100); Crazy paving pattern, 1 (100)		Bilateral, 1 (100)	40						
Marimuthu et al.	Unknown, 6 (100)	Unknown diagnosis method but reported as positive, 6 (100)										
Meyerowitz et al.	Nucleic acid-based testing, 36 (100)	Nucleic acid-based testing, 36 (100)										
Miyashita and Kuno	Nucleic acid-based testing, 161 (100)	Nucleic acid-based testing, 161 (100)										
Modi et al.	Nucleic acid-based testing, 1 (100)	Nucleic acid-based testing, 1 (100)				38.3						
Molina et al.	Nucleic acid-based testing, 8 (100)	No result of nucleic acid-based testing, 8 (100)										
Mondi et al.	Nucleic acid-based testing, 5 (60) Serological testing, 4 (40)	Nucleic acid-based testing, 3 (60); Serological testing, 4 (100)	GGO, 4 (57.1; Consolidation, 1 (14.3)	Focal, 1 (14.3)	Bilateral, 3 (42.9); Unilateral , 1 (14.3)						97	416.5
Nakamoto et al.	Nucleic acid-based testing, 1 (100)	Nucleic acid-based testing, 1 (100)	GGO, 1 (100)	Multifocal, 1 (100)		39.2	110	78	20	130	97	
Okoh et al.	Nucleic acid-based testing, 27 (100)	Nucleic acid-based testing, 27 (100)										
Parker et al. a	Nucleic acid-based testing, 1 (100)	Nucleic acid-based testing, 1 (100)	GGO, 1 (100); Consolidation, 1 (100)	Diffuse, 1 (100); Patchy, 1 (100)	Bilateral, 1 (100)				28		73	
Parker et al. b	Nucleic acid-based testing, 24 (100)	Nucleic acid-based testing, 24 (100)	Reticulation, 19 (79.2); Infiltrates, 9 (37.5)	Multifocal, 9 (37.5)	Bilateral, 19 (79.2)							

Patel and Pella	Nucleic acid-based testing, 1 (100)	Nucleic acid-based testing, 1 (100)				37	145	68	18	94	99	
Przydzial et al.	Nucleic acid-based testing, 2 (100)	Nucleic acid-based testing, 2 (100)	GGO, 1 (50); Infiltrates, 1 (50)		Bilateral, 1 (50)	37	141.5		17	80	88	
Qasim et al.	Nucleic acid-based testing, 1 (100)	Nucleic acid-based testing, 1 (100)				38.2	136	72	20	118	99	
Ridgway et a.,	Nucleic acid-based testing, 5 (100)	Nucleic acid-based testing, 5 (100)	GGO, 2 (40); Consolidation, 1 (20)	Perihilar, 2 (40); Patchy, 3 (60); Multifocal, 1 (20); Interstitial, 1 (20); Basal, 1 (20)	Bilateral, 2 (40); Unilateral , 1 (20)	37.8					94.4	
Riva et al.	Nucleic acid-based testing, 3 (100)	Nucleic acid-based testing, 3 (100)	Reticulation, 1 (33.3)	Interstitial, 3 (100)	Bilateral, 2 (66.7); Unilateral , 1 (33.3)	38.4						
Rivas et al.	Nucleic acid-based testing, 2 (100)	Nucleic acid-based testing, 2 (100)	Pleural empyema, 1 (50); Infiltrates, 1 (50)	Apical, 1 (50)	Bilateral, 1 (50); Unilateral , 1 (50)				31.5		96	
Ruan et al.	Nucleic acid-based testing, 4 (66.7); Serological testing, 2 (33.3)	Nucleic acid-based testing, 4 (100); Serological testing, 1 (50)	GGO, 4 (80); Consolidation, 2 (40); Reticulation, 3 (60)	Perihilar, 1 (20); subpleural, 1 (20); Peripheral, 3 (60); basal, 2 (40); Apical, 1 (20); Central, 1 (20)	Bilateral, 3 (60)						96	
Sasset et al.	Nucleic acid-based testing, 2 (100)	Nucleic acid-based testing, 2 (100)	Infiltrates, 2 (100)	Interstitial, 1 (50); Basal, 1 (50)	Bilateral, 1 (50); Unilateral , 1 (50)		120	70			<90	207.5
Shalev et al.	Nucleic acid-based testing, 31 (100)	Nucleic acid-based testing, 31 (100)										
Shekhar et al.	Nucleic acid-based testing, 5 (100)	Nucleic acid-based testing, 5 (100)				36.5	125	78.2	19	97.4	87.4	
Sigel et al.	Nucleic acid-based testing, 88 (100)	Nucleic acid-based testing, 88 (100)										
Stoeckle et al.	Unknown, 30 (100)	Unknown diagnosis method but reported as positive, 30 (100)	Infiltrates, 21 (70)		Unilateral , 21 (70); Bilateral, 21 (70)							

Su et al.	Nucleic acid-based testing, 1 (100)	Nucleic acid-based testing, 1 (100)	GGO, 1 (100)	Multifocal, 1 (100)	Bilateral, 1 (100)	37.8					98	
Sun et al.	Nucleic acid-based testing, 1 (100)	Nucleic acid-based testing, 1 (100)				37.2			20		100	
Suwanwongse and Shabarek a	Nucleic acid-based testing, 9 (100)	Nucleic acid-based testing, 9 (100)	GGO, 4 (44.4); Infiltrates, 4 (44.4)	Multifocal, 3 (33.3); Interstitial, 1 (11.1)	Bilateral, 8 (88.9)	38.6			28.8	126.8	87.4	
Suwanwongse and Shabarek b	Nucleic acid-based testing, 5 (100)	Nucleic acid-based testing, 5 (100)	GGO, 2 (40); Infiltration, 1 (20)		Bilateral, 3 (60)				32.7	107.5	87.8	
Toombs et al.	Nucleic acid-based testing, 3 (100)	Nucleic acid-based testing, 3 (100)				37.9	130	75.3	30.7	105	90.7	
Vizcarra et al,	Nucleic acid-based testing, 35 (100)	Nucleic acid-based testing, 35 (100)	Consolidation, 31 (88.6); infiltration, 20 (57.1)	Interstitial, 10 (28.6)	Bilateral, 20 (57.1)		93		18	92		462; <300, 5
Wang et al.	Nucleic acid-based testing, 1 (50), Serological testing, 1 (50)	Nucleic acid-based testing, 1 (100), Serological testing, 1 (100)				38.8	145	93	40	119	85	
Wu et al. a	Nucleic acid-based testing, 1 (50), Serological testing, 1 (50)	Nucleic acid-based testing, 1 (100), Serological testing, 1 (100)										
Wu et al, b	Nucleic acid-based testing, 2 (100)	Nucleic acid-based testing, 2 (100)	GGO, 2 (100)	Basal, 1 (50); Multifocal, 2 (100)	Bilateral, 2 (100)	39.5						
Yamamoto et al.	Nucleic acid-based testing, 5 (50), Serological testing, 5 (50)	Nucleic acid-based testing, 5 (100), Serological testing, 4 (80)	GGO, 4 (44.4)		Bilateral, 4 (44.4)							
Zhang et al.	Nucleic acid-based testing, 2 (50), Serological testing, 2 (50)	Nucleic acid-based testing, 2 (100), Serological testing, 2 (100)	GGO, 2 (50); Consolidation, 1 (25)	Peripheral, 2 (50); Basal, 1 (25); Multifocal, 2 (50)	Bilateral, 3 (75); Interlobar , 1 (25); Unilateral , 1 (50)	39.4						
Zhao et al.	Nucleic acid-based testing, 1 (50),	Nucleic acid-based testing, 1 (100),				37.2						

	Serological testing, 1 (50)	Serological testing, 1 (100)										
Zhu et al.	Nucleic acid-based testing, 1 (100)	Nucleic acid-based testing, 1 (100)				39			30		80	
Note: GGO=Ground glass opacities;												

Table S8: **Pharmacological treatment and supportive care given to HIV+COVID19 patients.**					
Author	**Number of patients whom ART regimen were maintained to treat complications due to COVID-19**	**Treatments given after diagnosis of COVID-19 or COVID-19 therapies**	**Number of patients requiring supplemental oxygen (nasal prong/face mask/high flow mask)**	**Number of patients requiring non-invasive mechanical ventilation (CPAP/BiPAP/NC/NRB)**	**Number of patients requiring invasive mechanical ventilation (Intubation/LMA/Extracorporeal membrane oxygenation)**
Adachi et al.,					
Altuntas et al.,					
Baluku et al.,		Azithromyxin (500mg daily for 5 days), hydroxychloroquine (400mg twice daily on day 3 for subsequent 5 days), paracetamol (1g three times a day for 5 days). Oral ciprofloxacin (500mg twice daily for 5 days) and oral rehydration salts were initiated (to treat gastrointestinal bacterial infection).			
Benkovic et al.,				1	
Blanco et al.,	Yes, 1 (20)			1	1
Boulle et al.,					
Byrd et al.,					
Calza et al., b		Darunavir/ritonavir or darunavir/cobicistat, 7; Hydroxychloroquine, 7; Azithromycin, 4; Enoxaparin, 3;			
Calza et al., a		Darunavir/ritonativir or darunavir/cobicistat, 12; rilprivirin, 5; Efavirenz, 1; Hydroxychloroquine, 13; Azithromycin, 6; Enoxaparin, 6;			
Chen et al.,	Yes, 1 (100)	Lopinavir/ritonavir (300/75mg, bid), interferon inhalation (5ug, bid)			
Chiappe et al.,		He was initially admitted for intraventidcular cyrptoccocoma, antifungal therapy with ampthotericin B deocycholate, fluconazole and waiting for ventricular-peritoneal shunt procedure. He was remain in Hospital while waiting for the procedure. High dose of corticosteroid was used when the pulmonary comprose worsen.		1	
Childs et al.,	Yes, 18 (100)	2 patients were switched to lopinavir/ritonavir, 2 patients treated with remdisivir; vasopressor, 5	11	3	4
Cipolat and Sprinz		Received antibiotics therapy with amoxicillin/clavulanate for 7 days. Hydroxychloroquine 400 mgbid plus azithromycin 500 mg		1	
Coleman et al.,		Treatment for PJP was commenced with intravenous cotrimaxazole (120 mg/kg/24H) and oral prednisolone 40 mg twice daily.		1	
Collins et al.,		Supportive care only, 9; hydroxychloroquine, 8; Hydroxychloroquine/azithromycine, 2 ; enrolled in remdesivir versus placebo trial, 1 . Average day of therapy given (5 days); Antibioitcs for secondary pneumonary to prevent community acquired coverage, 7; healthcare associated coverage, 4 (total antibiotics days for prevent		9	3

		secondary was 5 days			
Dandachi et al.,		Hydroxychloroquine, 67; azithromycin, 36; IL-6 antagonist (tocilizumab or sarilumab), 14; remdesivir, 12; convalescent plasma, 8; lopinavir/ritonavir, 6; vasopressor, 35	261	86	39
Del Amo et al.,					
Di Biagio et al., a	Yes, 4 (100)		1	2	
Di Biagio et al., b					
Di Giambenedetto et al.,	Modified	darunavir/cobicistat/emtricitabine/tenofovir alaenamide to replaced previous ART. Hydroxychloroquine, azithromycine. on 7 th after admission, sarilumad was administered (200mg intravenously), second dose on day 10. He was administered low molecular weight heparin at prophylactic dose.	1		
D'Ettorre et al.,	Yes, 1 (100)	Hydoxychloroquine 200mg bid and enoxaparin 400 Ul bid		1	
Elhadi et al.,		amoxicilline and clavulanate 875 mg every 12 h, prednisolone 40 mg every 12 h and mycolytic syrup to treat lobar pneumonia.		1	
Etienne et al.,					
Faranacci et al.,		Hydroxycholorquine and enoxaparin, Warfarin was replaced. Trimethoprim/sulfamethoxazole and steroid was started to treat Pneumocystis jirovecci without any ART.			
Gadelha et al.,				1	
Geretti et al.,					
Gervasoni et al.,		Paracetamol, 25 ; hydroxychloroquine, 8 ; azithromycin, 7; other antibiotics, 6; lopinavir/ritonavir, 5; tocilizumab, 2; remdesivir, 1	4		2
Gudipati et al.,		Antibiotics for community acquired pneumonia, 4; intrvenous fluids,3; systemic cortocosteroids, 3;	1		2
Guo et al.,					1
Haddad et al.,		He was started on cefepime, ampicillin, vancomycin, acyclovir for empiric bacterial meningitis and herpes encephalitis coverage *Herpes simplex. Hydroxychloroquine 400 mg twice a day for one day followed by 200 mg twice a day for four days in addition to azithromycin 500mg once followed by 250mg daily for 4 days. Then, treatment to treat Herpes was discontinued.			1
Hadi et al.,		Hydroxychloroquine, 25; azithromycine, 57; glucocorticoids, 52			
Harter et al.,		Boosted Darunavir, 33; PI containing regimen, 4		1	4
Ho et al.,		Hydroxychloroquine, 53; Steroid, 12; Azithromycin, 56; trial of investigational agent, 3		51	15
Hu et al.,		Glucocorticoids, 1	1	1	
Huang et al.,					
Inciarte et al.,	Yes, 53 (100)	21 received triple therapy with a combination of lopinavir/ritonavir, azithromycin, hydroxychloroquine. 2 (8) receive inteferon 1B, systemic glucocorticoids, 7 (28); tociluzimab, 8 (32)			3
Iordanou et al.,	Yes , 1 (100)	Levofloxacin 750 mg once daily to fight bacteria, oseltamivir 75 mg twice a day to			2

		prevent influenza A and B. azithrmycine 500 mg once daily, chloroquine 500 mg twice a day was given after COVID19 diagnosis was confirmed. Oseltamivir was stopped. Piperacilline tazobactam 4.5g four times a day, vancomycine 1750 mg loading dose followed by 100 mg three times a day to prevent hospital acquired pneumonia. Meropenem 2g three times a day, gentamicin 400 mg once daily, caspofungin 70 mg daily was administered to treat persistent fever.			
Isernia et al.,	Yes, 24 (100)	Lipinavir/ritonavir, 3; Hydroxycholoroquine, 2; Dexamethasone, 5; Toclizumab, 1			3
Karmen et al.,					5
Khaba et al.,		Intravenous stat dose of ceftriaxone and acetaminophen; timethoprim-sultamethoxazole 1920 mg 6hourly, hydrocortison 200mg 8 hourly, azithromycin 500 mg daily and enoxaparin 60mg daily.			
Kim et al.,	Yes, 1 (100)	ART maintained. Hydroxycholorquine 200 mg twice a day for 5 days.			
Kumar et al.,		mycophenolate 250 mg twice daily orally to treat covid19.			
Li et al.,				2	
Liu et al.,		Antiviral, 19; ART; 19; Corticosteroids, 1; Chinese herbal, 4; Immunoglobuline, 2	3		
Madge et al.,		None of them received treatment for COVID19	1		
Maggiolo et al.,					
Mang et al.,		Meropenem and linezolid as broad antibiotics regimen. Darunavir 600 mg twice daily, ritonavir 100 mg twice daily, tenofovir/emtricitabine 450/400 mg once daily. Ganciclovir 5 mg/kg . Intravenous trimethoprime sulfamethoxazole, 50 mg of prednisone daily to prevent adverse immune reaction in P. jirovecii and immune reconstitution inflammatory syndrome	1		1
Marimuthu et al.,	Yes, 5 (83.3)				
Meyerowitz et al.,			1		4
Miyashita and Kuno					19
Modi et al,.	Yes, 1 (100)	prednisone, tacrolimus, hydroxychloroquine for 5 days, ART continued	1		
Molina et al.,			3	1	
Mondi et al.,	Yes, 4 (80)		2		
Nakamoto et al.,		Hydroxychloroquine, 200mg twice a day for 14 days. After discharged, he was administered with bictegravir/emtricitabine/tenofovir alafenamide fumarate.	1		
Okoh et al.,	Yes, 27 (100)	Hydroxycholoroquine, 7; antibiotics (Azithromycine, ceftriaxone, doxycycline), 8; steroids, 1			
Parker et al., a	Changed	Lamivudine, zidovudine and lopinavir/ritonavir, amoxicillin clavulanic acid for severe community-acquired pneumonia; trimethoprim sulfamethoxazole for presume Pneumocystis pneumonia; chloroquine 650 mg daily for 2 days then 600 mg daily for 3 days			1
Parker et al., b					
Patel and Pella	Yes, 1 (100)	Emtricitabine 200 mg, tenofovir 25 mg every 24hours, atazanavir 300 mg every 24 hours, ritonavir, 100 mg every 24 hours; hydroxycholoroquine 400 mg every 12 hours for 1 day and 200 mg every 12h for 4 days. Oral azithromycine 500 mg every			

		24 hours for 7 days, zinc sulfate 220 mg every 8 hours for 5 days.			
Przydzial et al.,				3	
Qasim et al.		vancomycin, cefepime, metronidazole,fluconazole,topical miconazole			
Ridgway et al.,				2	
Riva et al.,	Yes, 1 (33.3)		1	2	1
Rivas et al.,				1	1
Ruan et al.,			1		
Sasset et al.,	Yes, 2 (100)				2
Shalev et al.,		None, 3; Hydrochloroquine, 24 ; Azithromycin, 16 ; Cortocosteroids, 8 ; IL-6 receptor inhibitor, 3. Remdesivir, 1		20	8
Shekhar et al.,	Yes, 5 (100)				2
Sigel et al.,		Hydroxychloquine, 67; Azithromycin, 66; Tocilizumab, 3; remdesivir or sarilumab, or anakinra, 4			
Stoeckle et al.,	Yes, 28 (93.3); No, 2	hydroxychloroquine, 20; corticosteroids, 4; remdesivir, 0; vasopressor, 4		15	4
Su et al.,	Yes , 1 (100), except Efavirenz	Lopinavir/ritonavir (400 mg/100mg twice/day), combined with interferon alpha inhalation, piperacillin/tazobactam, levofloxacin and oseltamivir. Efavirenz. was stopped. Arbidol 200 mg three times/day was added.			
Sun et al.,	Yes, 1 (100)	None			
Suwanwongse and Shabarek, a	Yes, 4 (44.4)				6
Suwanwongse and Shabarek, b	Yes, 2 (40)				3
Toombs et al.,	Yes, 2 (66.7)			2	2
Vizcarra et al.,		Hydroxycholoroquine, 30; Azithromycin, 15; Ritonavir-boosted lopinavir, 12; Tocilizumab, 4; Systemic corticosteroids, 13			5
Wang et al.,		Arbidol (0.2g tid); Methylprednisone 40mg/day for 5 days (corticosteroids). Moxifloxacin (antibacterial therapy); Sulbactam/cefoperazone was added for antibacterial therapy. Tocilizumab was given to fight the inflammation storm	1		
Wu et al., a	Yes, 1 (100)	Cefuroxime, traditional Chinese medicine (Lianqian oral solution and lianhua qingwen capsure). then it was changed to antiviral treatment Interferon atomization 5 million bid, ribavirin 150mg TID; abidol 200mg tid for antiviral treatment. Moxifloxacin 400 mg DQ to prevent bacterial infection.			
Wu et al., b	Yes, 1 (50)		2		
Yamamoto et al.,					
Zhang et al.,				1	
Zhao et al.,		Oseltamivir and interferon alpha inhalation			
Zhu et al.,		lopinavir/ritonavir (400/100 mg per dose twice daily for 12 days); methylprednisolone 0.8mg/kg once daily for 3 days through intravenous route. moxifloxacin 400 mg once daily for 7 days; y globulin 400 mg/kg once daily for 3	1	1	

		days			

Table S9: **Findings from studies regarding symptoms experienced by patients throughout clinical course.**

		Gastrointestinal manifestations					Neurological symptoms										Respiratory symptoms								Cardio symptoms		Musculoskeletal symptoms			Constitutional symptoms						
Author	Asymptomatic (Before diagnosis)	Abdominal discomfort/pain	Diarrhoea	Discomfort of the digestive tract	Loose stool	Nausea	Ageusia/	Agitation	Anosmia/inability to smell	Dizziness	Headache	Hypogeusia/	Hyposmia	Mental status changes/confusion	Seizure	Sinusitis	Cough with Expectoration/	Dry cough	Dyspnoea/	Haemoptysis/coughing up with blood	Polypnea or tachypnoea/	Nasal Congestion	Sore throat	Odynophagia	Chest pain/Chest tightness	Palpitation (Tachycardia)	Arthralgia	Backpain	Myalgia/	Chills	Decreased appetite/	Fever	Lethargy/	Malaise	Night sweats	Maculopapular rash
Adachi et al.,																																2				1
Altuntas et al.,			1															4	2		1											3		1		
Baluku et al.,			1								1										1				1				1		1					
Benkovic et al.,			1				1		1			1	1					2														3	3			
Blanco et al.,											3							5	3													5		3		
Boulle et al.,																																				
Byrd et al.,	1								5		9							6	9			2	2		1			3	8	2	2	9	4			
Calza et al., b																																				
Calza et al., a																		14	3										11			19	11			
Chen et al.,																		1														1				
Chiappe et al.,			1															1	1				1									1		1		
Childs et al.,																		13	12													11				
Cipolat and Sprinz		1	1			1						1	1					1	1										1		1	1				
Coleman et al.,																		1														1				
Collins et al.,			6			6	2		2		4							18	12				3		4				8	10		13		12		
Dandachi et al.,			63			67	33		33		69			21				205	146			37	56		54		128		128	198		198	140			
Del Amo et al.,																																				
Di Biagio et al., a																																				
Di Biagio et al., b	9																																			

Di Giambenedetto et al.,			1															1														1				
D'Ettorre et al.,																																1	1			
Elhadi et al.,											1							1	1													1				
Etienne et al.,																																				
Faranacci et al.,		1																	1													1				
Gadelha et al.,											1						1	1	1										1			1				
Geretti et al.,		13	25			23					18							88	84		50	8	13		24	51			26			94	40			
Gervasoni et al.,	1		7								3							23	10										4			41				
Gudipati et al.,			4				4		4									10	7													7				
Guo et al.,	3																	7	7													10	11			
Haddad et al.,		1	1			1		1						1	1			1								1						1	1			
Hadi et al.,																																				
Harter et al.,									6		7					6		25					7						7			22				
Ho et al.,			18						2		17			10				71	57			13	18						33			61				
Hu et al.,		6	7	6		4																									5	7				2
Huang et al.,																																				
Inciarte et al.,			16			4			15		28							45	10					11					27			42	25	29		
Iordanou et al.,																		1	1													1		1		
Isernia et al.,																																				
Karmen et al.,																																				
Khaba et al.,														1				1	1													1	1	1		
Kim et al.,							1		1								1	1				1	1						1	1						
Kumar et al.,							1		1									1				1				1				1		1				
Li et al.,										1								1	2						2						1	2	1			
Liu et al.,	4	1	1							1							5	13	4				1								1	9	2			
Madge et al.,																																				
Maggiolo et al.,	10						11		16									15	19													42	12			
Mang et al.,																		1	1													1				
Marimuthu et al.,	1																	2					1									5				
Meyerowitz et al.,			3	1	1	2	1		3		4			3				21	16			2	7						11	2	1	22	5	2	1	
Miyashita and Kuno																																				
Modi et al,.		1									1							1	1						1							1	1			
Molina et al.,																																				
Mondi et al.,	2																	3	1										1			3				
Nakamoto et al.,																		1														1				
Okoh et al.,			4			4												18	17										9			17	13			
Parker et al., a			1															1	1										1			1				

Parker et al., b		1	4				0		1		1							17	17	2			0		5				5			15		4		
Patel and Pella			1																												1			1		
Przydzial et al.,						1					1							1	2										1							
Qasim et al.											1							1					1							1		1			1	
Ridgway et al.,		1	3			2					2							3	4						1	2			1	2		4				
Riva et al.,																		2														3				
Rivas et al.,																		1	2		1											1	2	2		
Ruan et al.,																		4	3				1	1								4				
Sasset et al.,																		1	1		1					1						2		1		
Shalev et al.,																																23				
Shekhar et al.,		1	2						1			1						2	1						1				1	1		2	1			
Sigel et al.,																																				
Stoeckle et al.,		3	10			5	2		1		3						1	21	20			1			3				4			17				
Su et al.,										1								1														1				
Sun et al.,											1						1	1					1									1				
Suwanwongse and Shabarek, a			1			1												6	6			1							1			4				
Suwanwongse and Shabarek, b		1				1					1							4	2			2							2		1	4				
Toombs et al.,											1						1	2	2												1	2				
Vizcarra et al.,			10			3	4		4		8							24	21				7						13			25	21			
Wang et al.,																		1	1						1	1						1				
Wu et al., a																							1							1		1	1			
Wu et al., b			1														1	1	2				1						2			2	2			
Yamamoto et al.,							2											2					1									4				
Zhang et al.,										1							1		2			2	1		2	1					1	2	1		1	
Zhao et al.,																													1			1				
Zhu et al.,																		1	1													1				

Figure S1: Forest plot for pooled prevalence of asymptomatic PLHIV with COVID-19

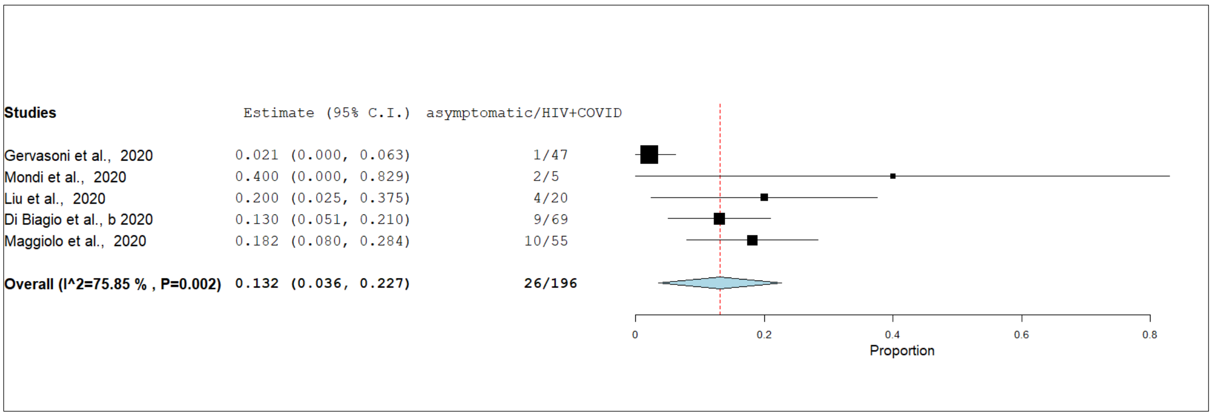

Figure S2: Forest plot for pooled prevalence of fever in PLHIV with COVID-19

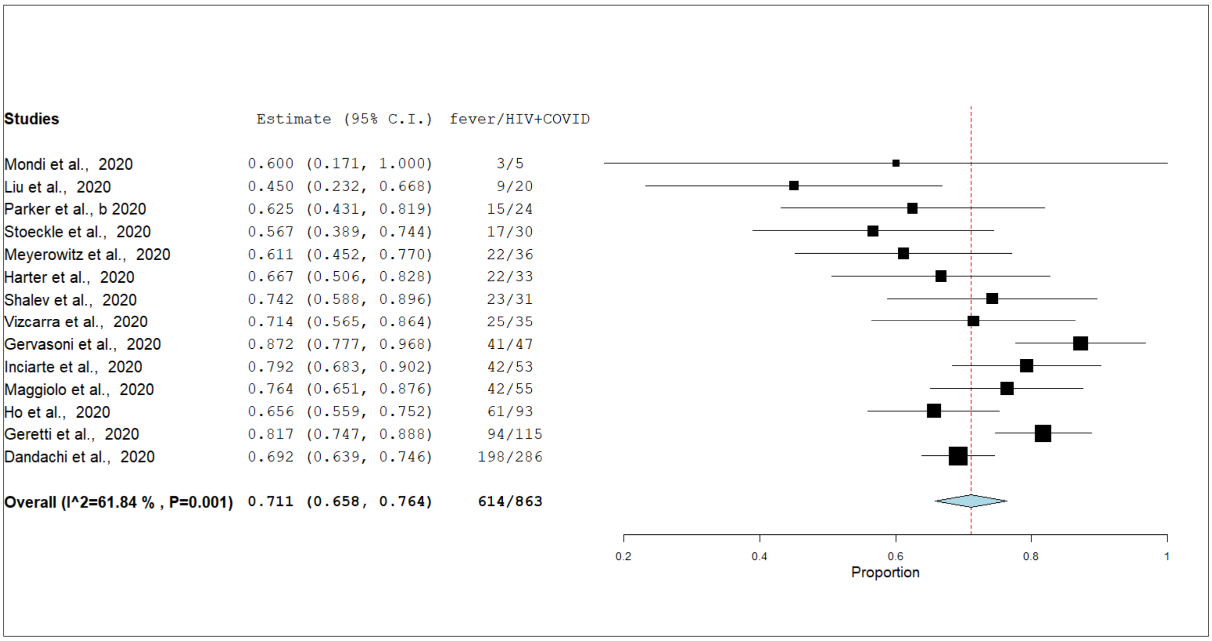

Figure S3: Forest plot for pooled prevalence of lethargy in PLHIV with COVID-19

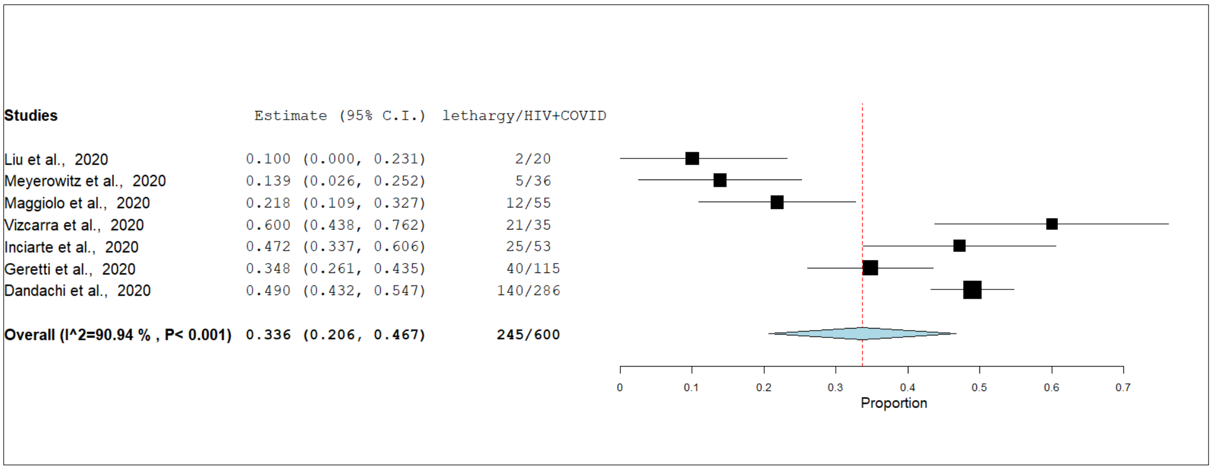

Figure S4: Forest plot for pooled prevalence of dry cough in PLHIV with COVID-19

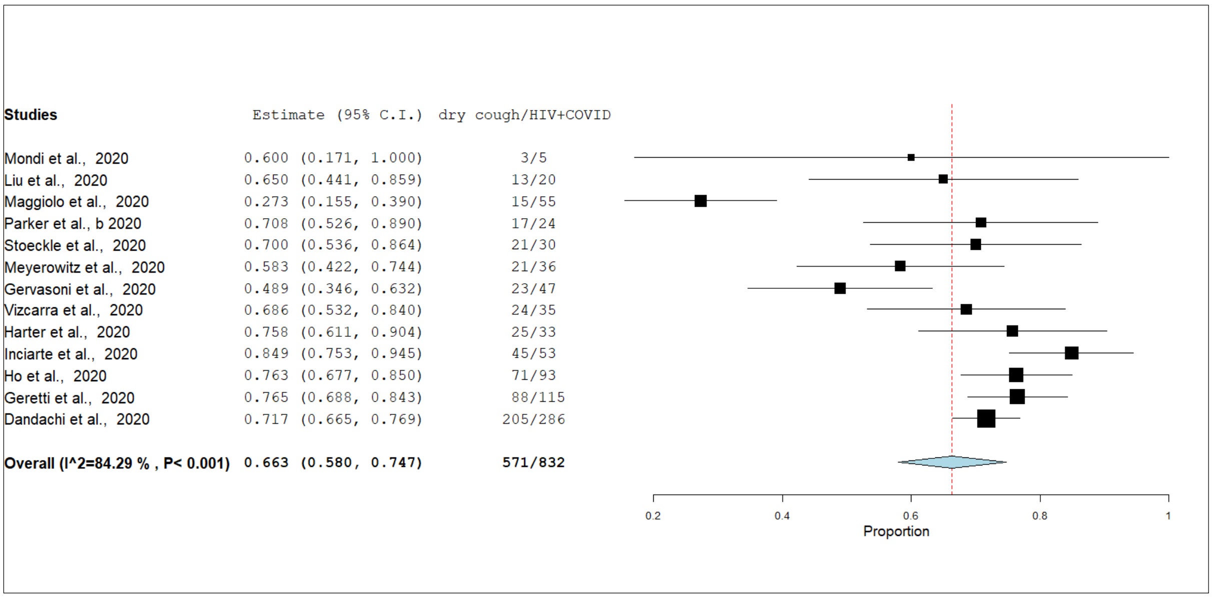

Figure S5: Forest plot for pooled prevalence of dyspnoea in PLHIV with COVID-19

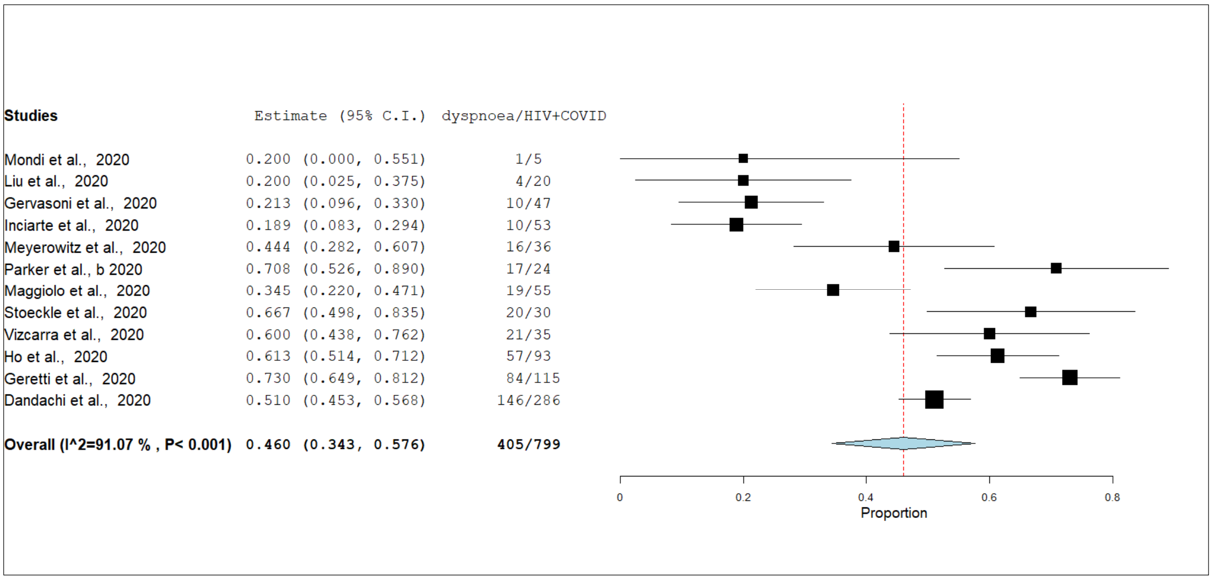

Figure S6: Forest plot for pooled prevalence of nasal congestion in PLHIV with COVID-19

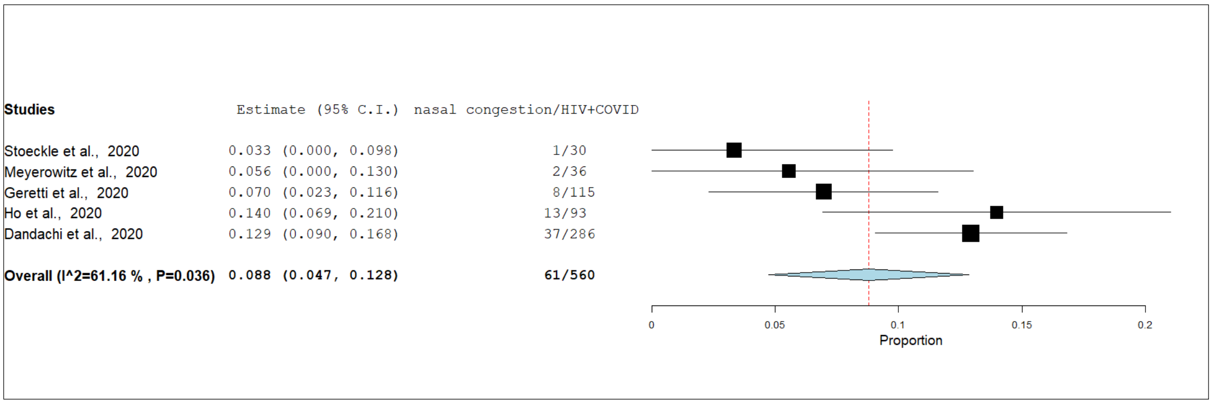

Figure S7: Forest plot for pooled prevalence of sore throat in PLHIV with COVID-19

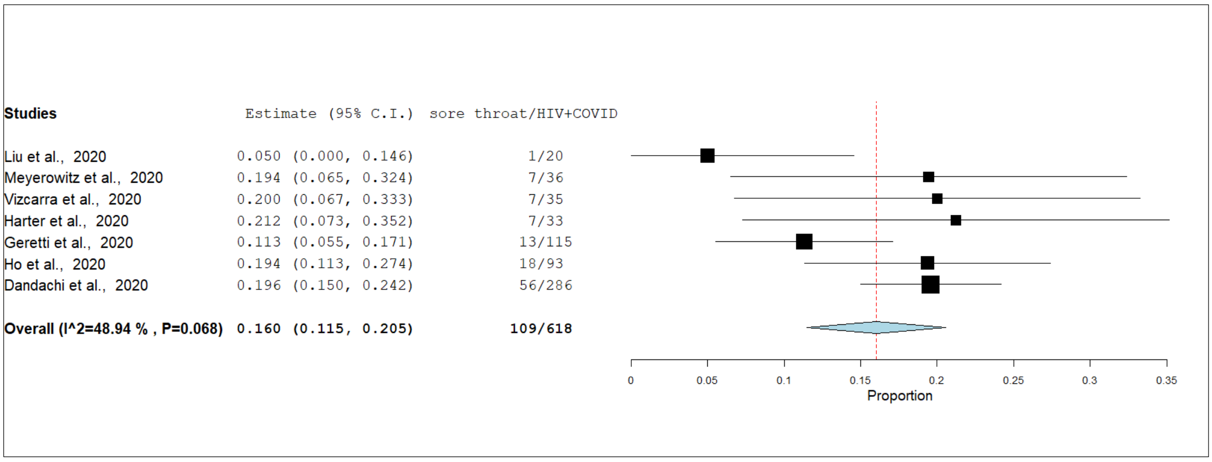

Figure S8: Forest plot for pooled prevalence of abdominal pain in PLHIV with COVID-19

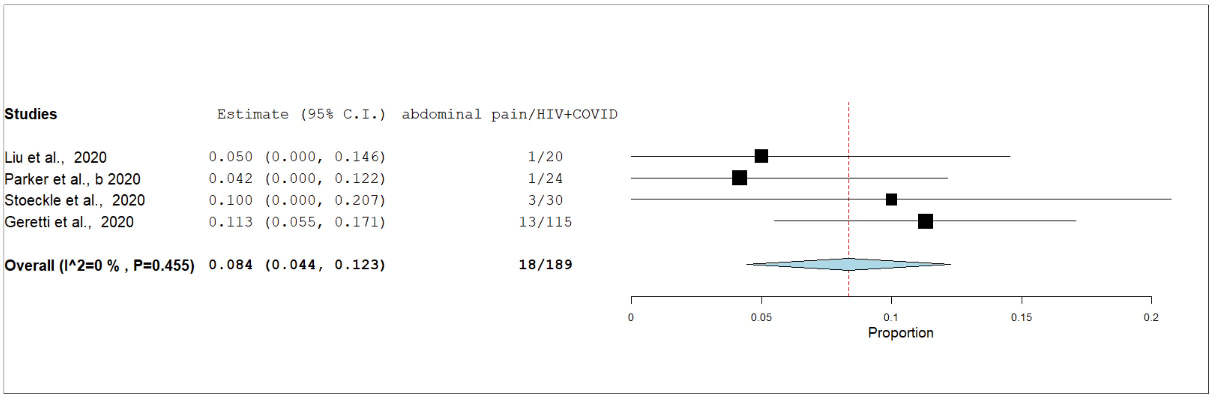

Figure S9: Forest plot for pooled prevalence of diarrhoea in PLHIV with COVID-19

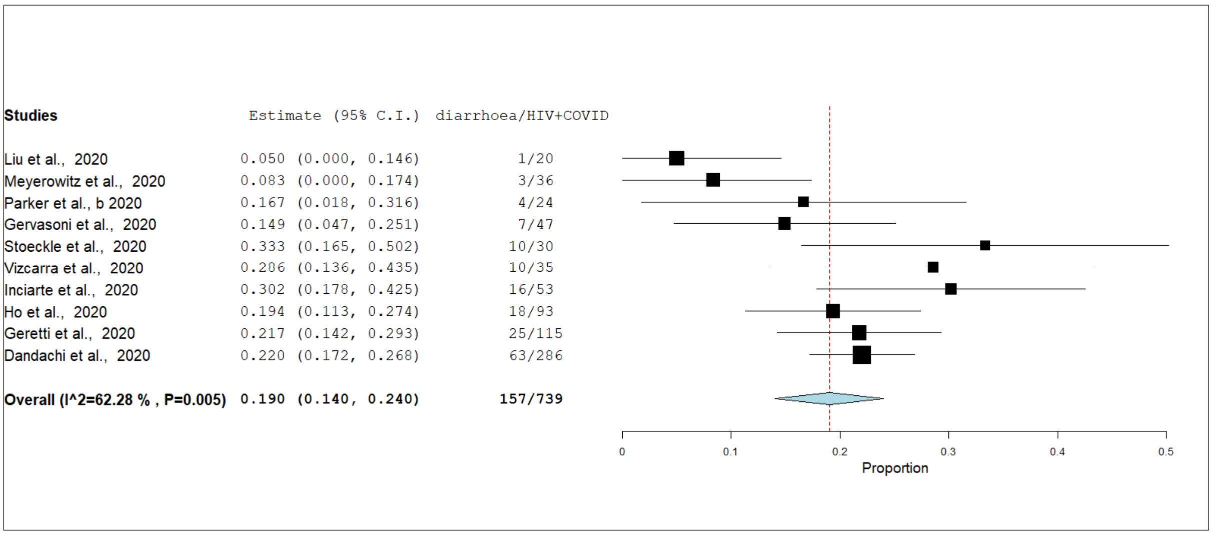

Figure S10: Forest plot for pooled prevalence of nausea in PLHIV with COVID-19

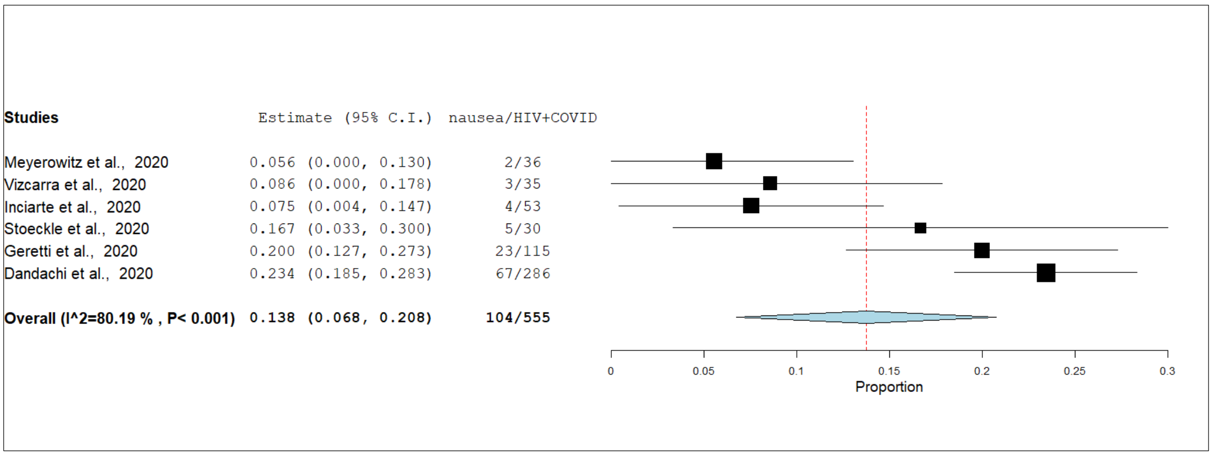

Figure S11: Forest plot for pooled prevalence of ageusia in PLHIV with COVID-19

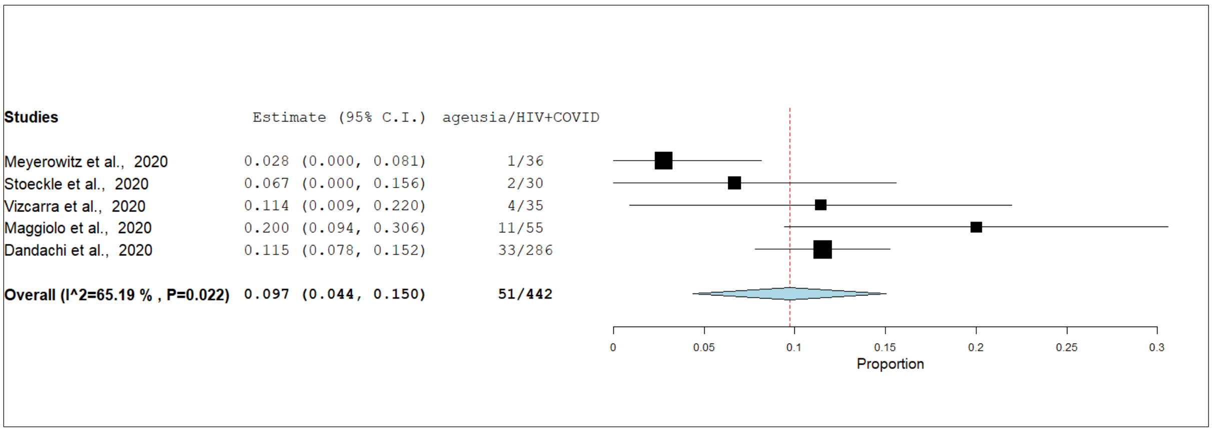

Figure S12: Forest plot for pooled prevalence of anosmia in PLHIV with COVID-19

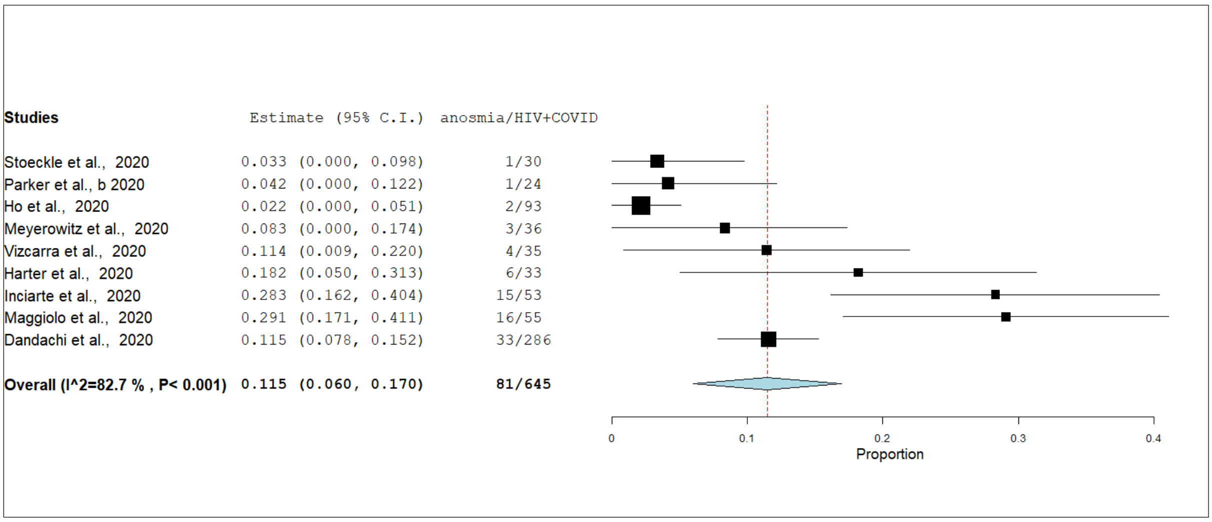

Figure S13: Forest plot for pooled prevalence of headache in PLHIV with COVID-19

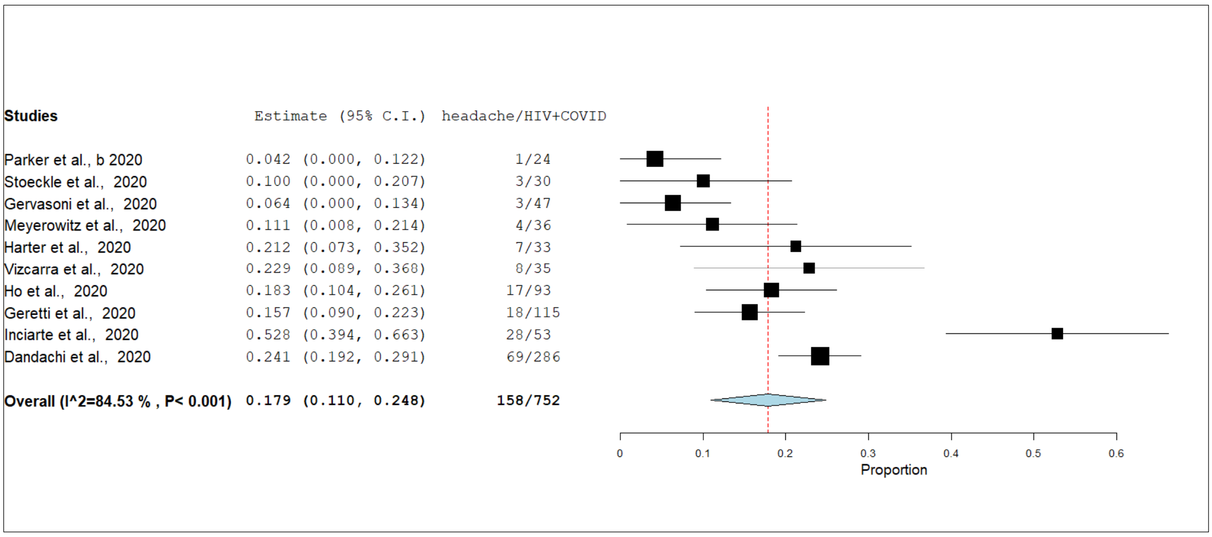

Figure S14: Forest plot for pooled prevalence of altered mental status/confusion in PLHIV with COVID-19

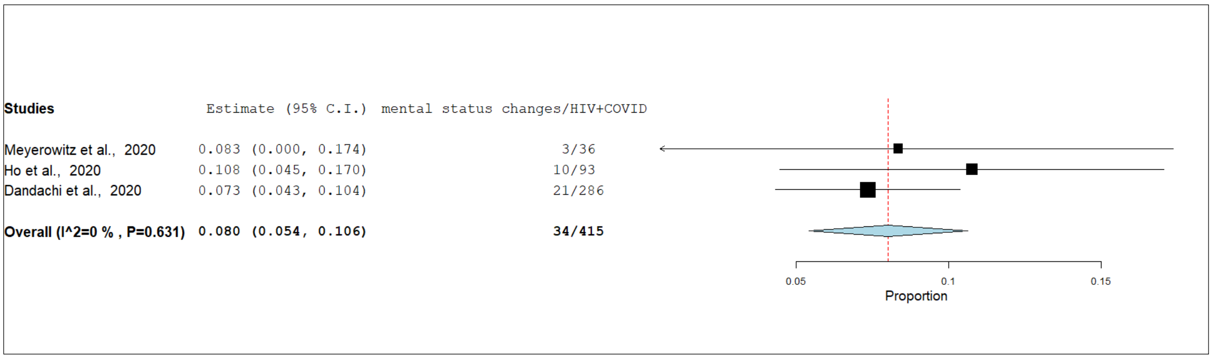

Figure S15: Forest plot for pooled prevalence of myalgia in PLHIV with COVID-19

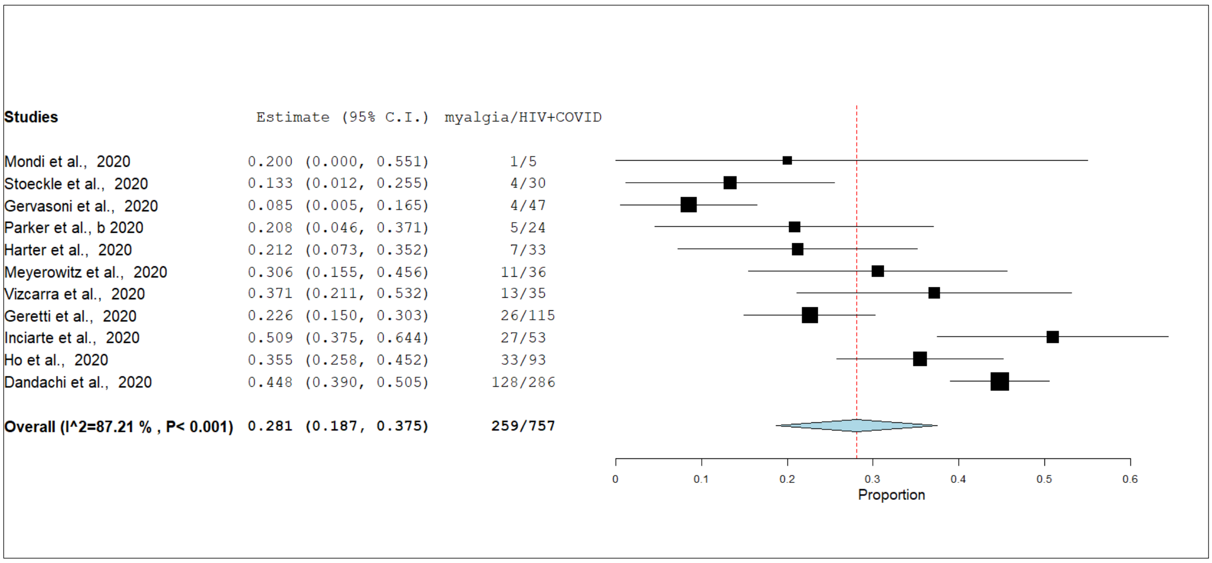

Figure S16: Forest plot for pooled prevalence of chest pain/chest tightness in PLHIV with COVID-19

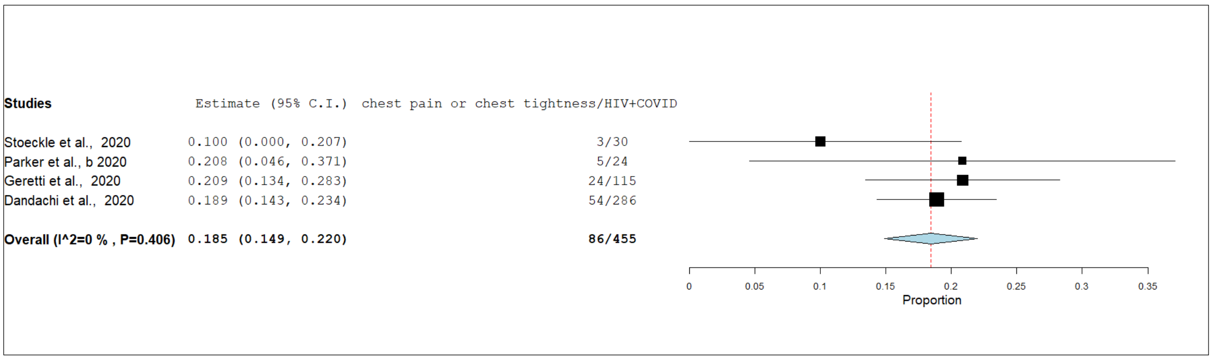

Table S10: Summary of estimate and its 95% CI for Pooled Incidence proportion of COVID-19 among PLHIV based on data accumulated from cohort studies using leave-one-out Meta-analysis forest plot.				
Studies	Estimate	95% CI	Standard error	p-values
Molina et al.	0.009	0.006, 0.011	0.001	<0.001
Huang et al.	0.009	0.006, 0.012	0.001	<0.001
Vizcarra et al.	0.008	0.005, 0.011	0.001	<0.001
Incarte et al.	0.009	0.006, 0.011	0.001	<0.001
Maggiolo et al.	0.007	0.005, 0.010	0.001	<0.001
Del Amo et al.	0.010	0.007, 0.012	0.001	<0.001
Boulle et al.	0.009	0.005, 0.013	0.001	<0.001

Figure S17: Leave-one-out Meta-analysis forest plot for pooled incidence proportion of COVID-19 among PLHIV based on data accumulated from cohort studies.

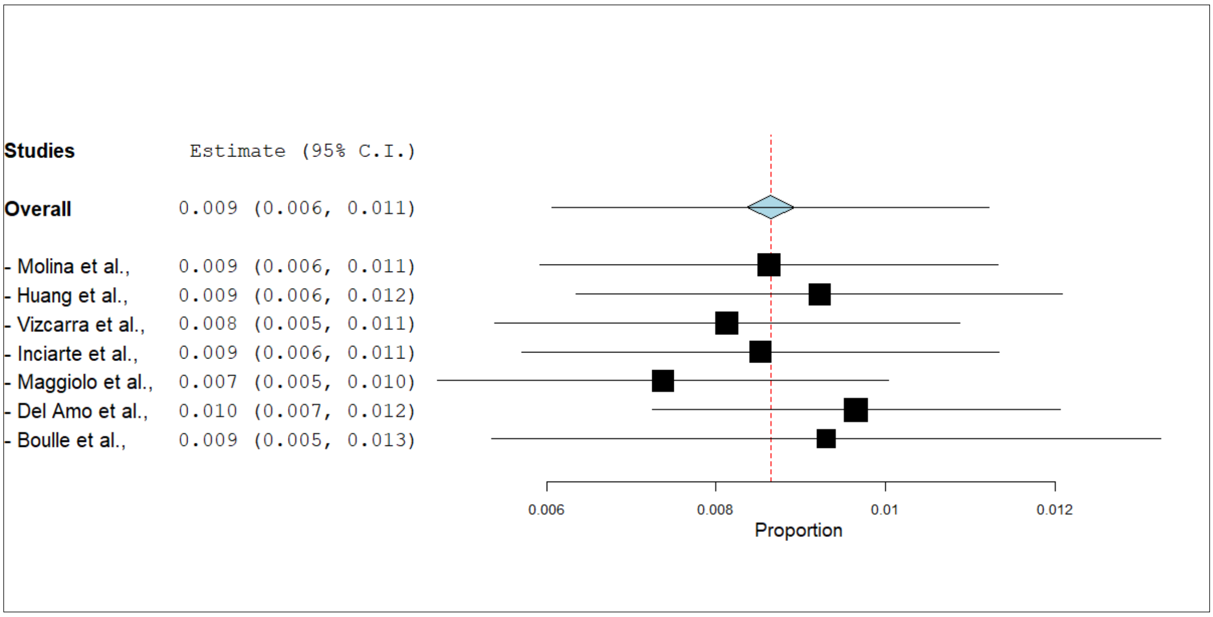

Supplement: Supplementary file 1 [file ijerph-18-03554-s001.pdf]
